# Pacing and placing in 161-km ultramarathons: Effects of sex and age

**DOI:** 10.1371/journal.pone.0322883

**Published:** 2025-05-12

**Authors:** Shawn E. Bearden, Irene van Woerden

**Affiliations:** 1 Department of Biological Sciences, College of Science and Engineering, Idaho State University, Pocatello, Idaho, United States of America,; 2 Department of Community and Public Health, College of Health, Idaho State University, Pocatello, Idaho, United States of America; University degli Studi di Milano, ITALY

## Abstract

Ultramarathons are growing in popularity, owing especially to the participation of women and masters athletes. Pacing strategy, which can vary by sex and age, is a critical variable in determining finishing place in races up to the marathon distance. Whether this is true in 161-km ultramarathons is unclear. We tested the hypotheses that pacing is a determining factor in finishing place and that pacing differs by sex and age in 161-km ultramarathons. Publicly available data from 161-km races (n = 6) were analyzed for years 2012–2022 (n = 56). Linear regression was used to analyze the proportion of time in each segment (between timing checkpoints) by place, sex, and age. In general, runners used the same percentage of their total race time in each segment independent of finishing place, sex, or age. The exception was that later finishers often ran proportionally faster at the start compared to earlier finishers. Finishing times increased with age but pacing was unaffected by age or sex. We conclude that slower finishers paced the same as faster finishers following a relatively quick start in these ultramarathons, and pacing was not consistently affected by sex or age. These findings should inform training decisions and racing strategy in 161-km ultramarathons.

## Introduction

One of the fastest growing endurance sports in the world is the ultramarathon, due in large part to increases in female and masters athlete participation [1]. Ultramarathons are defined as any running distance further than the marathon, which is 42.195 km (26.2 miles). The most common distances are 50 km [2], 80 km (aka 50-milers) [3,4], 100 km [5–7], 161 km (aka 100-milers) [8,9], and 320 km (aka 200-milers) [10] but also include multi-day races up to thousands of kilometers (e.g., Shri Chinmoy Self-Transcendence 3,100 Mile Race) [11–13]. The duration of these races typically exceeds 6 hours [14] and can last more than 48 hours or span many days or weeks [13,15]. The terrain varies from paved roads to rugged mountains with substantial elevation changes and variable weather.

Despite a clear understanding of the factors that predict performance in the marathon (V̇O_2_max, lactate turnpoint, running economy) [16,17], there is little consensus on the factors that predict performance in ultramarathons, especially as the distances increase [18–22]. Indeed, while most studies have reported that the physiologic predictors of performance in the marathon lose significance as race distances increase [18,20,22], there is evidence that V̇O2max and velocity at V̇O_2_max may still play a role in ultramarathon performance [23].

As with other endurance sports, completing an ultramarathon requires athletes to distribute their effort in a way that minimizes the rate of fatigue development and delays task failure through pacing [15]. Pacing is the pattern with which an athlete distributes effort across a race [24,25], and is believed to be an important determinant of “the extent to which individual potential is realized during athletic races” [26]. Finishers will place in order of average pace, but the pattern of pacing throughout the race may vary among runners [27]. The ways in which athletes of different abilities pace a race provides insight into the fatigue process and pacing strategy may be a critical factor in performance [27–29]. However, its role in determining finishing place in 161-km races has not been established.

In the 1995 100-km World Championships [30] and 2012–2013 100-km World Masters Championships [31], early finishers maintained a more consistent speed compared with later finishers who slowed down more over the duration of the race. Tan et al. [25] reported that the fastest runners slowed down less than later finishers at the Craze Ultramarathon 101-km race but there was no significant association between the rate of pace decline and finishing place for the 161-km race at the same event (2012 and 2013 editions). Thus, distances further than ~100 km may pose a different set of demands and require different pacing strategies. However, there have been no systematic analyses of the function of pacing in determining finishing place in 161-km ultramarathons. Moreover, ultramarathons take place in conditions that vary widely [13,15]. Yet there are no systematic comparisons of the potential pacing-placing interactions among races that vary in the many dimensions that characterize the sport (e.g., flat vs mountainous, smooth surfaces vs rocks/roots, loop vs repeated-lap vs point-to-point courses, altitude) or the extent to which sex or age alter pacing strategies in 161-km ultramarathons.

It is important to clarify the impact of sex and age on ultramarathon performance characteristics, in part, because it is the boom in participation by women and masters athletes that have primarily driven the exponential increase in ultramarathon events and finishers worldwide [1]. Men tend to be faster runners than women, but the differences narrow as race distance increases [3,10]. Women have paced more evenly than men in half-marathons and marathons [32,33] as well as at the 2012–2013 100-km World Masters Championships [31], whereas others have found no difference in pacing strategy between sexes for marathons or ultramarathons [34–36]. In the marathon, older runners start slower but also slow down less than younger runners with similar finishing times [37]. However, age was not significantly correlated with changes in pace in a 100-km [38] or 246-km [39] ultramarathon.

Understanding the factors that determine performance is important considering the > 10-fold increase in participation in ultramarathons over the past 25 years, according to the most comprehensive database of ultramarathons [12]. No studies have determined how finishers with different abilities pace 161-km ultramarathons, how pacing affects finishing place, and to what extent the relation between pacing and placing is influenced by characteristics of either the races or the finishers. Filling this gap in knowledge would substantially enhance the decision-making process in training and racing for athletes and coaches, while providing further insight into the relation between fitness and fatigue in ultramarathons [1]. Therefore, the purpose of this study was to test the hypotheses that pacing is a determining factor for finishing place in 161-km ultramarathons regardless of race characteristics and that finisher’s age and sex are critical factors influencing the relation between pacing and placing.

## Materials and methods

Ethical Approval. This study was approved by the Human Subjects Committee and Institutional Review Board of Idaho State University, with a waiver of the requirement for informed consent given that the study involved analysis of publicly available data.

Only a small fraction of races publish split times (the times each runner goes through check-points along the course), severely narrowing the options available for studying pacing throughout a race. In this study, rather than describing pacing in a single race, six 161-km races with widely varying characteristics were included so that general pacing patterns could be identified:

1] Hardrock Hundred Mile Endurance Run ® (HR) - single loop, Colorado, USA, 165 km, + 10,118 m, -10,118 m, average altitude 3,410 m, highest point 4,282 m, trail often rocky occasional cross-country sections, steep, held in July, morning start.
2] Hawaiian Ultra Running Team’s 100-mile Endurance Trail Run ® (HURT) - repeated lap, Hawaii, USA, 27.13 km per lap totaling 138 km, + 7,590 m, -7,590 m, average altitude 342 m, highest point 579 m, trail often rooted and muddy, hilly, held in January, morning start.
3] Rocky Racoon 100-mile Endurance Trail Run ® (RR) - repeated lap, Texas, USA, 31.53 km per lap totaling 158 km, + 1840 m, -1840 m, average altitude 99 m, highest point 116 m, trail fairly smooth but with some roots, flat to undulating, held in February, morning start.
4] Thames Path 100 ® (TP) - point-to-point, London, England, 161 km, + 1,691 m, -1,644 m, average altitude 34 m, highest point 82 m, gravel path, flat or mild undulating, held in May, morning start.
5] Ultra Trail du Mont Blanc ® (UTMB) - single loop, France-Italy-Switzerland, 170 km, + 10,510 m, -10,510 m, average altitude 1,635 m, highest point 2,573 m, trail often rocky, steep, held in August/September, evening start.
6] Western States Endurance Run ® (WS) - point-to-point, California USA, 160 km, + 5,518 m, -7,026 m, average altitude 1,215 m, highest point 2,648 m, trail generally smooth often dusty, hilly, held in June, morning start.

Race courses can differ in some years for various reasons including wildfires, heavy snow/rain, adding/removing an aid station, etc. The values given are from the published gpx (aka GPS Exchange Format) routes in 2022. As races did not always occur each year or data were not available, not all races have every year analyzed. A total of 56 races were analyzed (HR = 9, HURT = 7, RR = 11, TP = 10, UTMB = 9, WS = 10).

### Finisher demographics

Complete datasets were last collected from race reporting websites December 12, 2023 and saved to authors’ computers, including runners names, ages or birthdates, sex, split/segment times, and finishing times. Data from athletes who finished the 56 races were included in the analyses; data from participants who started but did not finish the race were excluded. For each race, sex and birth date are self-reported during participant registration.

### Time and proportion of time for each segment

Throughout races there are aid stations where runners check in for safety and timing. Runners use these aid stations to replenish food and drinks, change clothes, rest, and take care of related needs. When a runner reaches the aid station, the time is logged electronically (via chips in bibs or bracelets) or manually by dedicated race officials.

The proportion of time spent in each race segment (one timing-point arrival to the next timing-point arrival) was calculated by dividing the time spent in the race segment by the total race time for each finisher. Impossible datum points were excluded. For example, finishers who were recorded as completing a later segment prior to an earlier segment (e.g., finishing segment seven prior to finishing segment six) had that segment excluded from the analysis. In addition, segment times and distances for finishers whose proportion of time in a segment was substantially less than other finishers (> 3 SD faster than the mean proportion) were manually examined. Implausible segments - such as a slow runner recorded as running a segment 10X faster than the next fastest runner for that segment - were excluded from the analysis. Because runners in 161-km ultramarathons sometimes rest at aid stations or decide to walk long sections, long segment times were always considered plausible and left in the dataset. In total, 2% of datum points were excluded.

### Statistical analysis

T-tests were used to determine if there were significant differences at the bivariate level in finisher age and finishing time by sex for each race. To determine how consistent finisher placing was throughout each race, a correlation analysis was run on finisher placing at the end of each segment by finisher overall place.

To further discern the potential roles of finisher sex and age, a linear regression was used to predict the percentage of time spent in each segment by finisher overall place, sex, and age for each race. A few ultramarathons had a limited number of female finishers; while sex was included in the model for all races that it was available, the sex estimates for these races should be interpreted with caution. In addition, to determine if finisher sex and age were significantly associated with finisher overall place, a linear regression predicting finisher overall place by age and sex was run.

To determine if later finishers slowed down more, a generalized estimating equation model predicting pace by segment, finishing place, and the interaction between place and segment was run. Controls for sex and age were also included in the model. To determine if higher pace in the first segment(s) was resulting in a perceived overall decrease in pace, sensitivity tests were run. The same model as above, but excluding the first, and first and second, segments was run.

Given the large number of tests, statistical significance was defined as p < 0.001. All analyses were run in R (v 4.2.2).

## Results

A total of 56 races, and 23,207 competitors, across 6 ultramarathons were examined. The number of participants who finished the ultramarathons ranged from 46 (HURT, 2022) to 1,789 (UTMB, 2022) and the average finishing time ranged from 23.1 hours (TP, 2020) to 40.3 hours (UTMB, 2016) (Table 1). Overall, 13% of competitors were female. The UTMB races had the lowest proportion of females (8% female on average), while the RR races had the highest proportion of females (25% female on average). Finisher age was significantly different between males and females in two races (RR 2017; WS 2021); the mean age of males was higher than females in both cases (RR 2017: 44.6 ± 9.5 male, 39.1 ± 8.5 female and WS 2021: 45.2 ± 7.8 male, 39.4 ± 8 female). Finishing times were significantly different between males and females in 9 races (HURT 2020; RR 2014, 2015, 2017, 2018, 2019, 2021, 2022; TP 2017). For each of these races, the male mean finishing time was faster than the female mean finishing time.

**Table 1:** Summary of participant age (years) and finishing time (hours) by race and sex.

	Total			Female			Male			P.value	
Race	N	Mean (SD) Age	Mean (SD) Time	N	Mean (SD) Age	Mean (SD) Time	N	Mean (SD) Age	Mean (SD) Time	Age	Time
**HR**											
2012	98	43.5 (9)	38.2 (6)	12	45 (8.7)	39.3 (5.7)	86	43.3 (9.1)	38.1 (6.1)	0.532	0.491
2013	104	46.3 (9.3)	39.4 (5.8)	11	46.5 (8)	39.9 (5.9)	93	46.2 (9.5)	39.3 (5.8)	0.907	0.760
2014	100	46.5 (9.1)	39.5 (6.1)	11	47.5 (7.4)	42.6 (5)	89	46.3 (9.4)	39.2 (6.1)	0.629	0.054
2015	123	44.1 (8.4)	39.4 (6.1)	17	43.9 (6.9)	39.5 (5.9)	106	44.1 (8.6)	39.4 (6.2)	0.911	0.927
2016	114	45 (9.4)	40 (5.9)	14	44 (8.4)	40.4 (7.3)	100	45.1 (9.6)	39.9 (5.7)	0.653	0.791
2017	126	45 (9.4)	38.5 (6.2)	18	44.9 (9.2)	39 (6.6)	108	45 (9.5)	38.4 (6.1)	0.972	0.728
2018	114	46.4 (8.8)	39.8 (5.8)	11	46.5 (10.2)	39.7 (6.3)	103	46.4 (8.7)	39.9 (5.8)	0.989	0.932
2021	112	46.9 (8.5)	39.7 (6.1)	11	46.8 (10.3)	39.2 (6.4)	101	46.9 (8.3)	39.8 (6)	0.975	0.778
2022	115	45.3 (8.5)	39.5 (6)	19	43.3 (7.5)	39.4 (4.9)	96	45.7 (8.7)	39.5 (6.2)	0.238	0.906
**HURT**											
2015	60	37.7 (8.5)	31.3 (3.9)	9	36 (8.2)	31.9 (3)	51	38 (8.6)	31.2 (4)	0.519	0.553
2016	53	40.2 (8.6)	31.6 (3.9)	9	40.3 (5)	33.6 (1.7)	44	40.1 (9.2)	31.2 (4)	0.920	0.008
2017	54	40.5 (7.8)	32 (3.7)	14	38.9 (7.7)	33.7 (2.3)	40	41.1 (7.9)	31.4 (3.9)	0.375	0.013
2018	78	40.3 (9.4)	32.4 (3.6)	24	39 (9.4)	33.7 (2.8)	54	40.9 (9.4)	31.8 (3.7)	0.431	0.017
2019	69	39.5 (9.3)	31.5 (3.6)	14	35.1 (8.4)	32.8 (2.4)	55	40.7 (9.3)	31.1 (3.9)	0.043	0.057
2020	63	39.9 (8.5)	31.8 (3.7)	19	39.8 (7.8)	34.1 (1.7)	44	39.9 (8.9)	30.8 (3.9)	0.949	**<0.001**
2022	46	39.7 (7.9)	32.6 (3.1)	13	37.5 (5.4)	33.4 (2.5)	33	40.6 (8.7)	32.3 (3.3)	0.157	0.225
**RR**											
2012	217	43 (9.8)	24.8 (3.7)	42	40.9 (9.5)	26 (2.9)	175	43.5 (9.8)	24.6 (3.8)	0.114	0.010
2013	229	41.6 (9.5)	24.9 (3.6)	62	39.1 (8.9)	25.8 (3.1)	167	42.5 (9.6)	24.6 (3.7)	0.012	0.016
2014	280	42.9 (9.8)	25 (3.9)	85	40.5 (9)	26.1 (3.5)	195	44 (9.9)	24.5 (4)	0.004	**0.001**
2015	249	41.6 (9.2)	24.3 (4.2)	63	40.7 (8.5)	25.8 (3.8)	186	41.9 (9.4)	23.8 (4.2)	0.328	**0.001**
2016	242	43.7 (9.6)	25.2 (3.7)	62	42.7 (9.5)	26.5 (3.3)	180	44 (9.6)	24.8 (3.7)	0.325	0.001
2017	210	43.3 (9.6)	24.7 (3.8)	52	39.1 (8.5)	26 (3.1)	158	44.6 (9.5)	24.3 (3.9)	**<0.001**	**0.001**
2018	201	42.9 (9.3)	25.6 (3.4)	51	41.7 (10.1)	27 (3)	150	43.2 (9)	25.1 (3.4)	0.350	**<0.001**
2019	207	41.6 (8.9)	25.6 (3.4)	51	41.5 (8.9)	27.1 (2.6)	156	41.7 (8.9)	25.1 (3.5)	0.881	**<0.001**
2020	251	42.8 (9.3)	25.5 (3.4)	63	41.4 (9)	26.3 (2.7)	188	43.3 (9.4)	25.2 (3.6)	0.156	0.014
2021	252	43 (8.9)	24.4 (3.8)	55	41.1 (7.9)	26 (3.4)	197	43.6 (9.1)	24 (3.7)	0.049	**<0.001**
2022	199	41.6 (9.7)	25.6 (4)	50	40.7 (8.9)	27 (3.3)	149	41.9 (10)	25.1 (4)	0.443	**0.001**
**TP**											
2013	90	40.6 (7.9)	24.9 (3.2)	10	36.6 (10.7)	25.2 (3.2)	80	41.1 (7.4)	24.8 (3.2)	0.224	0.738
2014	147	41.9 (7.7)	23.4 (2.7)	24	40.1 (7.4)	24.1 (2)	123	42.2 (7.7)	23.3 (2.8)	0.208	0.096
2015	182	42.1 (8.5)	23.7 (2.8)	24	41.3 (8.6)	24.4 (2.7)	158	42.2 (8.5)	23.6 (2.8)	0.631	0.221
2016	207	42.6 (8.2)	23.3 (2.9)	24	41.4 (8.7)	24.3 (2.9)	183	42.7 (8.1)	23.2 (2.9)	0.482	0.103
2017	209	43 (7.5)	23.6 (2.9)	34	42.9 (7.2)	25.7 (2.4)	175	43 (7.6)	23.2 (2.9)	0.935	**<0.001**
2018	182	43.2 (8.4)	24.1 (2.6)	32	42.3 (7.2)	25 (2.5)	150	43.5 (8.6)	23.9 (2.6)	0.422	0.038
2019	225	44.2 (7.3)	23.5 (2.9)	41	43.2 (7.7)	24 (3)	184	44.4 (7.2)	23.4 (2.9)	0.376	0.255
2020	183	43.9 (7.7)	23.1 (3)	24	44.3 (8.1)	24.3 (2.8)	159	43.8 (7.7)	22.9 (3)	0.778	0.038
2021	187	44.9 (7.1)	23.2 (3.2)	23	45.3 (6.3)	24.3 (2.1)	164	44.8 (7.3)	23.1 (3.3)	0.711	0.018
2022	203	46.1 (8.9)	23.5 (2.9)	43	45.3 (7.7)	23.8 (2.8)	160	46.4 (9.2)	23.5 (3)	0.430	0.455
**UTMB**											
2013	1686	42.7 (8.0)	39.5 (5.3)	140	42.5 (8.0)	40.8 (5.1)	1546	42.8 (8.0)	39.4 (5.3)	0.678	0.003
2014	1582	42.2 (7.9)	39.8 (5.3)	114	42.0 (8.1)	40.7 (5.7)	1468	42.2 (7.9)	39.7 (5.3)	0.733	0.066
2015	1631	42.3 (7.8)	39.9 (5.2)	132	41.4 (7.5)	40.5 (5.3)	1499	42.4 (7.8)	39.9 (5.2)	0.178	0.198
2016	1468	41.6 (7.3)	40.3 (5.0)	131	41.3 (7.3)	41.0 (5.3)	1337	41.6 (7.3)	40.3 (5.0)	0.638	0.137
2017	1687	41.9 (7.3)	39.3 (5.5)	147	40.7 (7.3)	39.8 (5.5)	1540	42.0 (7.3)	39.3 (5.5)	0.039	0.292
2018	1778	42.7 (7.6)	39.9 (5.4)	168	41.8 (7.3)	40.2 (5.9)	1610	42.8 (7.6)	39.9 (5.3)	0.079	0.493
2019	1556	42.7 (7.7)	39.7 (5.4)	145	41.9 (7.9)	40.4 (6)	1411	42.8 (7.7)	39.6 (5.4)	0.211	0.112
2021	1521	44.4 (7.7)	40.1 (5.5)	111	42.5 (7.8)	40.0 (6.5)	1410	44.6 (7.7)	40.1 (5.4)	0.008	0.827
2022	1789	43.8 (7.7)	39.0 (5.8)	138	43.4 (8.0)	39.6 (6.3)	1651	43.8 (7.7)	39.0 (5.7)	0.627	0.238
**WS**											
2012	317	40.9 (8.7)	24.7 (3.9)	52	39.1 (7.4)	24.3 (3.8)	265	41.3 (8.9)	24.7 (3.9)	0.065	0.477
2013	277	41.2 (9)	25.6 (3.4)	52	39.4 (8.6)	25.7 (3.4)	225	41.6 (9)	25.6 (3.5)	0.107	0.870
2014	296	41.5 (8.6)	24.8 (3.8)	55	39.5 (8)	25.8 (3.7)	241	41.9 (8.7)	24.6 (3.8)	0.054	0.034
2015	254	42.1 (8.6)	25.4 (3.7)	53	41.9 (9.2)	26.2 (3.5)	201	42.1 (8.5)	25.2 (3.8)	0.900	0.070
2016	254	41.9 (8.5)	25.2 (3.8)	54	41.2 (7.7)	25.8 (3.5)	200	42.1 (8.7)	25 (3.9)	0.475	0.172
2017	248	40.8 (8.3)	26.1 (3.4)	46	37.9 (8.6)	25.9 (3.3)	202	41.5 (8.1)	26.1 (3.4)	0.013	0.718
2018	248	40.8 (8.4)	24.4 (3.5)	49	38.4 (8)	24.1 (3.5)	199	41.4 (8.4)	24.5 (3.5)	0.022	0.459
2019	321	42.7 (8.3)	25.2 (3.8)	67	40.1 (8.1)	25.5 (4.1)	254	43.4 (8.3)	25.2 (3.8)	0.004	0.517
2021	208	43.9 (8.2)	26.3 (3.7)	48	39.4 (8)	25.2 (4.3)	160	45.2 (7.8)	26.7 (3.5)	**<0.001**	0.039
2022	305	43.4 (8.1)	25.8 (3.7)	69	41.3 (8)	25.9 (4)	236	44 (8.1)	25.8 (3.6)	0.017	0.884

### Correlation analysis

Place at the end of each segment was significantly associated with that participant’s overall finishing place (all correlations p < 0.001; Table 2). The correlation with finishing place at the end of the first segment ranged from 0.60 (HURT 2020) to 0.86 (TP 2017), indicating there is already high predictive value (strong to very strong correlation) for finishing place by the end of the first segment of a race.

**Table 2:** Correlation between final finishing place and finishing place at each segment.

Race	S1	S2	S3	S4	S5	S6	S7	S8	S9	S10	S11	S12	S13	S14	S15	S16	S17	S18	S19	S20	S21	S22	S23	S24	S25
**HR**																									
2012	0.82	0.87	0.87	0.91	0.92	0.93	0.94	0.95	0.97	0.99	0.99	1	1												
2013	0.79	0.86	0.87	0.89	0.91	0.91	0.93	0.95	0.96	0.96	0.98	0.99	1	1											
2014	0.77	0.8	0.87	0.88	0.89	0.90	0.91	0.93	0.95	0.95	0.97	0.97	0.98	1											
2015	0.85	0.91	0.92	0.93	0.93	0.94	0.93	0.93	0.96	0.97	0.97	0.98	0.99	1	1										
2016	0.76	0.79	0.80	0.81	0.82	0.83	0.88	0.90	0.95	0.95	0.97	0.98	0.99	1											
2017	0.82	0.88	0.89	0.90	0.92	0.92	0.94	0.95	0.96	0.97	0.98	0.99	0.99	1	1										
2018	0.72	0.79	0.85	0.86	0.86	0.88	0.91	0.93	0.96	0.96	0.98	0.99	0.99	1											
2021	0.76	0.84	0.86	0.86	0.86	0.90	0.89	0.91	0.92	0.93	0.94	0.97	0.99	1	1										
2022	0.81	0.85	0.88	0.88	0.89	0.89	0.9	0.91	0.93	0.94	0.96	0.97	0.99	1											
**HURT**																									
2015	0.68	0.73	0.78	0.83	0.86	0.85	0.89	0.91	0.92	0.94	0.95	0.96	0.98	0.99	1										
2016	0.62	0.66	0.7	0.69	0.74	0.78	0.82	0.85	0.88	0.92	0.96	0.97	0.98	0.99	1										
2017	0.81	0.86	0.87	0.88	0.87	0.88	0.90	0.91	0.91	0.93	0.95	0.96	0.97	0.98	1										
2018	0.67	0.71	0.75	0.77	0.83	0.85	0.88	0.90	0.95	0.97	0.98	0.98	0.98	0.99	1										
2019	0.69	0.76	0.79	0.82	0.83	0.85	0.87	0.89	0.92	0.94	0.95	0.97	0.98	0.99	1										
2020	0.60	0.72	0.76	0.81	0.85	0.88	0.89	0.91	0.92	0.94	0.94	0.96	0.98	0.99	1										
2022	0.71	0.76	0.81	0.82	0.78	0.81	0.86	0.87	0.91	0.92	0.92	0.95	0.98	0.99	1										
**RR**																									
2012	0.76	0.85	0.90	0.96	1																				
2013	0.81	0.88	0.91	0.96	1																				
2014	0.85	0.91	0.94	0.97	1																				
2015	0.83	0.88	0.92	0.97	1																				
2016	0.78	0.85	0.92	0.97	1																				
2017	0.78	0.86	0.92	0.97	1																				
2018	0.77	0.87	0.95	1																					
2019	0.78	0.86	0.94	1																					
2020	0.80	0.88	0.94	1																					
2021	0.78	0.87	0.92	0.97	1																				
2022	0.79	0.85	0.9	0.96	1																				
**TP**																									
2013	0.61	0.77	0.87	0.91	0.92	0.91	0.95	0.97	0.97	0.99	1														
2014	0.75	0.88	0.92	0.96	0.99	1																			
2015	0.81	0.81	0.89	0.91	0.95	0.99	1																		
2016	0.84	0.9	0.91	0.95	0.99	1																			
2017	0.86	0.91	0.93	0.95	0.99	1																			
2018	0.66	0.80	0.84	0.93	0.97	1																			
2019	0.65	0.81	0.85	0.89	0.96	0.99	1	1																	
2020	0.63	0.82	0.89	0.91	0.94	0.98	1	1																	
2021	0.80	0.86	0.88	0.89	0.91	0.93	0.97	0.98	0.99	0.99	1														
2022	0.68	0.78	0.84	0.90	0.92	0.93	0.95	0.96	0.97	0.98	1	1													
**UTMB**																									
2013	0.70	0.73	0.79	0.82	0.83	0.84	0.87	0.88	0.89	0.89	0.90	0.91	0.92	0.93	0.94	0.95	0.96	0.97	0.97	0.98	0.98	0.99	1	1	
2014	0.71	0.74	0.79	0.82	0.84	0.85	0.87	0.88	0.89	0.89	0.90	0.91	0.92	0.93	0.94	0.95	0.96	0.97	0.97	0.98	0.99	0.99	1	1	
2015	0.68	0.71	0.76	0.80	0.82	0.83	0.85	0.87	0.88	0.89	0.89	0.91	0.92	0.92	0.93	0.94	0.96	0.97	0.97	0.98	0.99	0.99	1	1	
2016	0.68	0.71	0.76	0.79	0.80	0.82	0.84	0.86	0.87	0.87	0.88	0.89	0.90	0.91	0.92	0.93	0.94	0.96	0.96	0.98	0.98	0.99	1	1	
2017	0.66	0.70	0.75	0.79	0.81	0.82	0.85	0.86	0.87	0.88	0.88	0.90	0.91	0.91	0.92	0.93	0.94	0.96	0.97	0.97	0.98	0.99	1	1	
2018	0.69	0.72	0.78	0.83	0.84	0.85	0.88	0.89	0.90	0.90	0.91	0.92	0.93	0.93	0.94	0.94	0.95	0.96	0.97	0.97	0.98	0.99	0.99	1	1
2019	0.61	0.76	0.81	0.84	0.86	0.86	0.88	0.89	0.90	0.91	0.91	0.92	0.93	0.93	0.94	0.95	0.96	0.97	0.98	0.98	0.99	1	1	1	
2021	0.72	0.76	0.81	0.84	0.86	0.86	0.88	0.89	0.90	0.90	0.91	0.92	0.93	0.94	0.95	0.96	0.97	0.98	0.98	0.99	0.99	1	1		
2022	0.73	0.76	0.81	0.85	0.85	0.87	0.89	0.90	0.90	0.91	0.91	0.93	0.94	0.95	0.95	0.96	0.97	0.98	0.98	0.99	1	1	1		
**WS**																									
2012	0.82	0.84	0.86	0.88	0.88	0.91	0.92	0.92	0.93	0.94	0.94	0.96	0.97	0.98	0.98	0.99	1	1	1	1					
2013	0.65	0.73	0.78	0.79	0.8	0.83	0.84	0.87	0.89	0.90	0.92	0.94	0.96	0.97	0.98	0.99	0.99	1	1						
2014	0.79	0.82	0.86	0.88	0.9	0.91	0.92	0.93	0.94	0.95	0.95	0.97	0.98	0.98	0.99	0.99	1	1	1	1					
2015	0.82	0.83	0.86	0.89	0.89	0.91	0.91	0.93	0.93	0.94	0.95	0.96	0.98	0.98	0.99	0.99	0.99	1	1						
2016	0.79	0.83	0.85	0.87	0.89	0.90	0.90	0.93	0.94	0.95	0.96	0.97	0.97	0.98	0.99	0.99	1	1							
2017	0.75	0.74	0.80	0.84	0.84	0.85	0.87	0.89	0.90	0.91	0.92	0.93	0.95	0.95	0.97	0.98	0.99	1	1						
2018	0.70	0.79	0.80	0.84	0.86	0.86	0.87	0.89	0.91	0.92	0.93	0.94	0.96	0.97	0.98	0.99	1	0.99	1						
2019	0.83	0.86	0.87	0.89	0.90	0.91	0.92	0.93	0.94	0.95	0.96	0.98	0.99	0.99	1	1	1								
2021	0.75	0.76	0.80	0.83	0.85	0.86	0.87	0.89	0.90	0.91	0.94	0.95	0.97	0.97	0.98	0.99	0.99	1	1						
2022	0.78	0.8	0.82	0.85	0.87	0.89	0.89	0.90	0.92	0.93	0.94	0.95	0.95	0.96	0.97	0.97	0.96	0.99	1	1	1				

### Pacing analysis of all races

**Fig 1:**
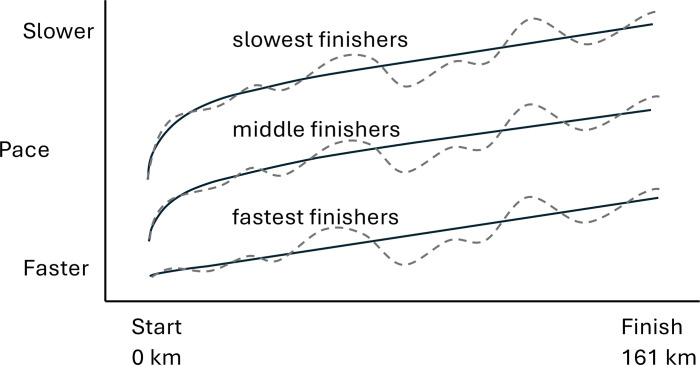
Summary of generalized pacing-placing findings. While all finishers slowed down over the duration of the races, slower finishers started their races fast relative to their overall performance. Finishers paced their races similarly after the first ~30 km of the race. Solid lines depict a flatter course where pace is less variable, and the dashed lines depict a mountainous course where pace fluctuates substantially due to significant elevation changes. These generalized findings were true regardless of sex or age.

In 51 of 56 races, the percentage of total race time spent in the first segment declined with later finishing (Fig 1 and Table S1). The percentage of total race time spent in the second segment also declined with later finishing in 27 of 56 races.

A few races presented consistent associations between pacing and placing across years for a few segments, indicating something meaningful about those segments. For instance, finishers who placed later in the race consistently spent a greater percentage of their time in segment 4 of RR (i.e., 4^th^ of 5 ~32 km laps) significant for all 8 of the 11 races that had 5 segments), segment 12 of WS (~100 km into race, significant effect in 10 of 10 races), and segments 16–19 of UTMB (~140–170 km into race, significant effect for 9 of 9 races).

For the UTMB races, males often spent a lower proportion of time in segments 2 (~ 20 km into race, 3 races), 6 (~42 km into race, 4 races), and 16 (~120 km into race, 4 races) and a greater proportion of time in segments 4 (~38 km into race, 4 races) and 12 (~88 km into race, 3 races) than females. Other than a few other race segments, the percentage of time spent in a segment was not associated with participant sex (11 other segments).

Age was a significant predictor of the percentage of time spent in a segment for many of the UTMB races. Notably, older participants often spent a greater percentage of time in the earlier segments (~8–50 km into race, segments 2, 3, 5 and 6, age was significant in 30 of the 36 segments). Other than the UTMB races, only six other race segments had significant associations between participant age and percentage of time spent in a segment. The statistical significance in these cases is unlikely to be meaningful because the effect sizes are very small. For instance, for UTMB 2022, the model predicts the percentage of time in segment to change from 3.98 to 4.02 for a 40 vs 50-year-old male who finishes 500th (3.98 + 10*0.0043 = 4.02), which is a difference of 54.7 seconds in a 38-hour finish time.

### Finisher overall place: Sex and age

**Fig 2:**
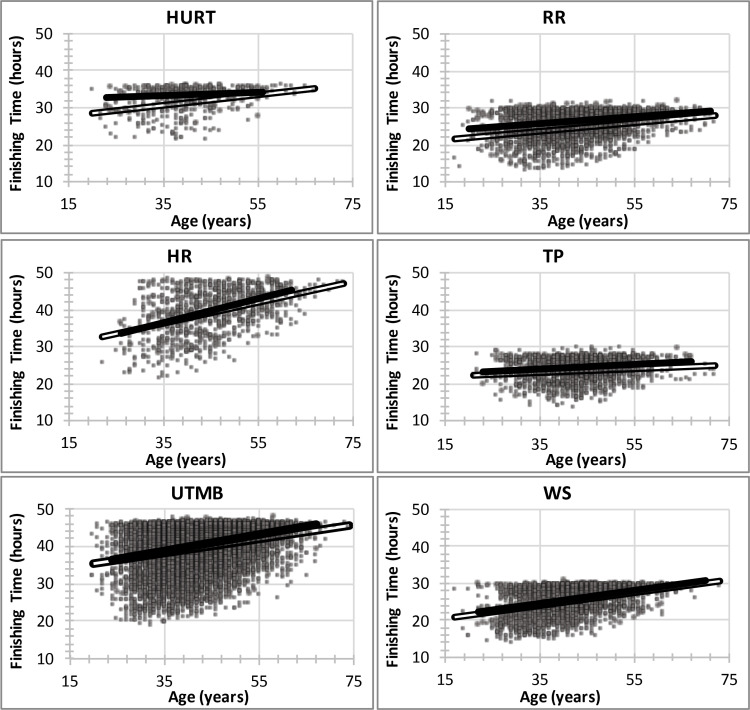
Finishing time increases with age. Although pacing did not differ by age, older finishers had slower average pace than younger finishers. For ease of presentation, all years of each race are pooled in respective figures; open squares and trendlines are males and filled circles and trendlines are females. Significant age-time relations (p < 0.001) were found for all years in HR, UTMB, and WS; 2020 in HURT; 2012-2017, 2019, 2021 in RR; but none of the years in TP. See Table S2 for specific beta and p values. The flat ‘ceiling’ on data in each graph is due to the cut-off times imposed by each event, beyond which participants are no longer allowed to continue or be counted as official finishers. RR = Rocky Raccoon, HR = Hardrock, TP = Thames Path, WS = Western States.

Finisher overall place was significantly associated with sex for 13 of the 56 races examined (Table S2); for all 13 races, males were faster than females. The beta estimate was negative for 39 of the remaining races, indicating males tended to be faster than females. Finisher overall place was significantly associated with age for 37 of the 56 races; finishers placed later as age increased (Fig 2).

### Pacing changes in the 28 lap-based or flat races of HURT, RR, and TP

Results of the generalized estimating model relating pace and place are in Table S3. Participants slowed down over the duration of each race (pace in minutes/km increased). The interaction term between place and segment was significant for 20 of the 28 races analyzed, each time with a positive beta-value. The beta-value is an interaction term that indicates, after controlling for place and segment, later finishers *slowed down* significantly more than earlier finishers in those races. However, upon removing the first and second segments, where later finishers systematically started fast, only five races showed a pattern of pace change by placing (RR 2019, TP 2016–2018, TP 2022) and, for three of those, the later finishers progressively *sped up* significantly more across the remaining segments (RR 2019, TP 2016–2017).

There was no significant association between pacing and placing by sex or age, after controlling for the other variables in the model.

## Discussion

We tested the hypotheses that pacing is a determining factor for finishing place in 161-km ultramarathons and that participant’s sex and age influence the relation between pacing and placing. These hypotheses were largely refuted by the data. The first major finding was that later finishers spent proportionally less time in the first segment (51 of 56 races), and sometimes second segment (25 of 56 races) of the race. Thus, the slower the runner the faster they began their race relative to their abilities; slower runners start too fast. The second major finding was that all finishers paced the remaining 80–85% of the race similarly and there was no relation between pacing and placing. Thus, after an exuberant start, slower runners did not pace differently than faster runners and did not slow down more than faster runners. These findings are illustrated in Fig 1. The third major finding was that neither sex nor age affected pacing in general. Athletes who finished the race in similar times were found to pace the race the same regardless of sex or age. And the fourth major finding was that older runners were generally slower than younger runners overall (Fig 2) but, again, athletes who finished the race in similar times were found to pace the race the same regardless of age (Table S2).

Slower finishers systematically and proportionally started races faster than their abilities, based on finishing times. Later finishers also start faster in the marathon, relative to finishing times, than earlier finishers [40] and in shorter ultramarathons [41]. This may be due to an over-exuberance of less fit or less experienced participants. There may also be a tethering effect whereby slower runners attempt to keep up with the relatively faster runners at the start. It is only once the field has separated somewhat, and perhaps as an early sense of fatigue develops, that slower runners adopt a pace commensurate with their abilities. Specifically, slower runners ran the first 21 ± 12 km too fast in 91% of the races analyzed and continued to run too fast for another ~16 km in 45% of the races. Thus, slower runners should consider being especially cautious for roughly the first 20–40 km in 160-km races and slowing down more than comes naturally.

Our analysis focused only on finishers, and we did not assess pacing patterns among non-finishers. It is possible that some runners, particularly those at risk of missing cutoff times, may have started at an even more aggressive pace relative to their ability, ultimately leading to their early fatigue. Future research should explore how pacing strategies differ between finishers and non-finishers, particularly in relation to cut-off times and race attrition.

In ultramarathons shorter than 161-km, differences in pacing between faster and slower runners have been reported. At the 1995 100 km World Championships [30], faster runners started faster and maintained speed for longer than slower finishers. Likewise, after analyzing results at the 2011 World Masters Athletics Ultra Championships, Renfree et al. [31] reported that the top half of finishers in each age category consistently ran at lower percentages of their average race speed than the bottom half of finishers during the initial stages of the race. Tan et al. [25] reported that the fastest runners slowed down significantly less than later finishers at the Craze Ultramarathon 101-km race (2012 and 2013 editions); however, there was no significant difference in the rate of pace decline across finishing place for the 161-km race at the same race. Our results corroborate the results of Tan et al. [25] at the 161-km distance and extend them beyond a single race, to encompass 161-km races of widely varying characteristics. Collectively, these previous studies and our current analyses have indicated that pacing is a feature of placing in races up to approximately 100 km but not in longer races. Our results take a broad view of all participants in each race. Pacing strategies may become more relevant to specific placing within a small range of places, which can be meaningful to the individual athlete even if not statistically significant. Whether a runner overtakes the person ahead of them or whether they are overtaken by the one behind may require subtle and nuanced competitive pacing strategies that are not within the scope of this study [29,42].

In 13 of the 56 races, female finishers were significantly slower overall than male finishers but there was no significant difference between male and female average finishing times in the other 43 races. As explained in the 2023 consensus statement of the American College of Sports Medicine [43], athletic performance is significantly influenced by biological sex through the inherent variances in anatomy and physiology, regulated by sex chromosomes and hormones; consequently, in sports that emphasize endurance, muscle strength, speed, and power, males typically surpass females by 10%-30%. Reasons for males outperforming females in ultramarathons have been discussed in depth elsewhere [44,45]. While the top male may usually outperform the top female in ultramarathons, the gap between male and female ultramarathon finishers diminishes with longer distances/durations [43] and was eliminated in 80% of the 161-km races analyzed for this study. Moreover, pacing was not consistently different as a function of sex. The notion that women pace more evenly than men was not supported by any of the races analyzed in this study.

Age of peak performance for most finishers of ultramarathons occurs in one’s 40s, but up to 10 years younger for the top finishers [11,46,47]. Declines in endurance performance with age appear to be determined primarily by declining maximal stroke volume, heart rate and arterio-venous O(2) difference, blood O(2) carrying capacity, skeletal muscle capillary density and oxidative capacity though most of these can be mitigated through lifelong endurance training with the exception of maximal cardiac output via decreasing maximal heart rate [48,49]. Indeed, older finishers were generally slower than younger finishers in the races we analyzed but this appeared to be course-dependent (Fig 2). Regardless, the primary and novel finding this study was designed to investigate is that older finishers paced the same as younger finishers of similar abilities. Thus, as runners grow older, they may or may not slow down depending on the course, but finishing time does not appear to be a function of differences in pacing.

The best road-marathon performances are limited by V̇O_2_peak, the O_2_ cost of running (economy), and the lactate turn-point [16,17]. While ultramarathon race performances are associated with these variables, the strength of the associations decline with increasing distance and the consensus among studies is that they fail to provide significant predictive value in races longer than ~100 km [18–22]. For example, in ~ 50 km races, V̇O2peak and the running velocity at V̇O_2_peak were associated with performance [18,22]. At 80–100 km, only running velocity at V̇O _2_peak was associated with performance [18,21]. Beyond 100 km, the same laboratory-based measures failed to predict performances [18,20,22] with the exception of an association with the velocity at V̇O _2_peak found in [22] but not in [18].

Because laboratory tests predicted performance in a 68-km race but not in a 121-km race, Gatter et al. [20] concluded that laboratory-based metrics are determinants of performance in races up to ~12 hours duration but not at ~ 28 hours. In 161-km races, Coates et al. [18] found no significant correlations between performance and velocity at V̇O2max (vV̇O2max), V̇O_2_max, caloric cost of running, oxygen cost per kilometer, resting heart rate, blood pressure, body mass index, or training volume (past month, past year, strength training, or total hours training). However, others have reported a significant correlation with performance for both V̇O_2_max and vV̇O_2_max at the UTMB 171-km race, the latter having the strongest correlation [23]. The reasons for this discrepancy among studies remain unclear and further research is needed. The idea, however, is consistent with our findings that overall speed (such as vV̇O_2_max) predicted finishing place while pacing or the rate of pace decline, as a proxy for performance durability, did not.

Although extensive research is aimed at informing the training of long-distance runners, there is much yet to be learned about best practices [50], particularly for races exceeding 100 km. Some ideas can be drawn from our findings because they provide evidence that an athlete’s “durability” - the capacity to sustain physiological capacities over time - is not a determining factor in finishing place at the 161-km distance. Given the lack of significant correlation between many traditional laboratory-based physiological measures and performance, perhaps it is not surprising that finishers of 161-km ultramarathons of all abilities pace their races similarly after the starting 20–40 km. If earlier finishers were more *durable* and if *durability* were a distinguishing factor in performance at this distance, then we would expect earlier finishers to slow down less, which was not the case. This does not mean that performance capacity did not deteriorate during these races. Indeed, the overall decline in pace for all finishers is evidence there is appreciable fatigue. Rather, the deterioration was proportionally similar among all those capable of completing the race and did not deteriorate in a fashion that compromised performance in slower runners any differently than in faster runners.

Ultramarathons are performed in a wide range of conditions that vary in geographic location, surface terrain, weather, and other variables [13,15,51]. Because a 161-km ultramarathon covers a lot of territory and may last multiple days, runners also experience extremes in conditions within a single race such as freezing temperatures on windy mountain ridges at nighttime and hot conditions exposed to the sun in valleys during daytime [52–55]. Moreover, runners may have different experiences, especially in mountainous 161-km races where weather varies with time of day, altitude, geographic location, and local microclimates [56,57]. We attempted to control for these widely varying conditions by including races with many characteristics so that any features robust to those factors would retain significance across the 56 races studies. Indeed, our findings have substantial generalizability because the major outcomes were consistent among races. Nevertheless, it may be fruitful in future studies to examine environmental conditions to determine how factors such as altitude, running surface, temperature, wind, rain, etc. may impact more subtle strategies in pacing. Local data collection, perhaps on each runner’s person, would be required for the most valid analyses of such factors.

We conclude that it is not a matter of running faster later in the race or maintaining an early pace for longer that distinguishes finishing place but rather relative speed at all points, equally. This conclusion is supported by analyses made by others where performances in ultramarathons correlate with personal best times in shorter races and running speed during training [58,59] but not with training volume [18]. Based on this finding, athletes and coaches would benefit more from training focused on improving sustainable speed (e.g., maximal steady-state and critical speed) rather than training to enhance fatigue-resistance as such. Nevertheless, we recognize there is likely to be utility in performing long training days to develop other aspects of the craft of ultramarathon running like testing gear, improving nutrition and hydration strategies, practicing footcare, or developing mental stamina and resilience. These recommendations are the same regardless of sex or age.

## Supporting information

S1 Table Linear model results predicting the proportion of total race time spent in each segment. (DOCX)

S2 Table Linear model results predicting finishing place by sex and age. (DOCX)

S3 Table Generalized Estimating Equation results predicting pace by place. (DOCX)
