# Supplementary material for: Pacing and placing in 161-km ultramarathons: Effects of sex and age
Source: PLoS One. 2025 May 12;20(5):e0322883. doi: 10.1371/journal.pone.0322883 (PMC12068597; doi:10.1371/journal.pone.0322883)
Supplement: S1 Table — (DOCX) [file pone.0322883.s001.docx]

Supplementary Table 1: Linear model results predicting the proportion of total race time spent in each segment.

| **Race** | S1 | S2 | S3 | S4 | S5 | S6 | S7 | S8 | S9 | S10 | S11 | S12 | S13 | S14 | S15 | S16 | S17 | S18 | S19 | S20 | S21 | S22 | S23 | S24 | S25 |
| --- | --- | --- | --- | --- | --- | --- | --- | --- | --- | --- | --- | --- | --- | --- | --- | --- | --- | --- | --- | --- | --- | --- | --- | --- | --- |
| **HR2012** |  |  |  |  |  |  |  |  |  |  |  |  |  |  |  |  |  |  |  |  |  |  |  |  |  |
| (Intercept) | 8.52** | 5.87** | 8.99** | 6.46** | 1.9** | 3.56** | 9.04** | 6.21** | 15.32** | 11.6** | 5.01** | 8.75** | 9.08** |  |  |  |  |  |  |  |  |  |  |  |  |
| I(Place/10) | -0.16** | -0.06** | -0.08* | -0.02 | 0.02 | 0.03 | 0.09 | 0.05 | -0.07 | -0.18** | -0.04 | 0.07 | 0.35** |  |  |  |  |  |  |  |  |  |  |  |  |
| Sex: Male | -0.24 | -0.17 | -0.04 | -0.04 | -0.08 | -0.03 | -0.25 | -0.03 | -0.24 | -0.06 | 0.2 | 0.25 | 0.67 |  |  |  |  |  |  |  |  |  |  |  |  |
| Age | 0.02* | 0.01 | 0 | 0 | 0 | 0.01 | 0 | 0 | 0.01 | -0.01 | -0.01 | -0.04 | -0.02 |  |  |  |  |  |  |  |  |  |  |  |  |
| **HR2013** |  |  |  |  |  |  |  |  |  |  |  |  |  |  |  |  |  |  |  |  |  |  |  |  |  |
| (Intercept) | 7.07** | 6.1** | 3.28** | 6.35** | 14.73** | 8.71** | 5.47** | 8.75** | 5.24** | 3.99** | 13.12** | 8.68** | 6.35** | 2.28** |  |  |  |  |  |  |  |  |  |  |  |
| I(Place/10) | -0.12** | -0.05* | -0.03** | -0.05** | 0.02 | 0.17* | 0.14** | -0.02 | -0.11** | -0.05 | -0.07 | 0.03 | 0.05 | 0.09* |  |  |  |  |  |  |  |  |  |  |  |
| Sex: Male | -0.12 | -0.12 | -0.06 | 0.02 | 0.3 | 0.1 | -0.12 | -0.05 | 0.01 | -0.14 | -0.08 | -0.07 | 0.05 | 0.27 |  |  |  |  |  |  |  |  |  |  |  |
| Age | 0.01 | 0 | 0 | 0 | -0.04* | -0.05* | 0.01 | -0.01 | 0 | 0.01 | -0.01 | 0.01 | 0.03* | 0.04** |  |  |  |  |  |  |  |  |  |  |  |
| **HR2014** |  |  |  |  |  |  |  |  |  |  |  |  |  |  |  |  |  |  |  |  |  |  |  |  |  |
| (Intercept) | 8.43** | 5.37** | 7.31** | 6.35** | 2.06** | 3.83** | 9.23** | 5.63** | 13.62** | 3.14** | 11.33** | 5.46** | 8.36** | 9.78** |  |  |  |  |  |  |  |  |  |  |  |
| I(Place/10) | -0.18** | -0.05* | -0.01 | 0.02 | 0.03 | 0.02 | 0.1 | 0.05 | -0.12 | -0.03 | -0.08* | -0.06** | 0.02 | 0.3** |  |  |  |  |  |  |  |  |  |  |  |
| Sex: Male | -0.07 | 0.05 | 0.51* | 0.63* | 0.07 | 0.06 | 0.42 | -0.24 | -0.22 | -0.02 | -0.38 | -0.13 | -0.09 | -0.58 |  |  |  |  |  |  |  |  |  |  |  |
| Age | 0.02* | 0.01 | 0.01 | -0.01 | 0 | 0 | -0.01 | 0.02 | 0 | 0 | 0 | -0.01 | -0.02 | 0 |  |  |  |  |  |  |  |  |  |  |  |
| **HR2015** |  |  |  |  |  |  |  |  |  |  |  |  |  |  |  |  |  |  |  |  |  |  |  |  |  |
| (Intercept) | 7.14** | 6.07** | 3** | 6.22** | 2.77** | 9.89** | 6.14** | 4.56** | 8.25** | 5.6** | 4.79** | 14.39** | 9.74** | 7.65** | 3.92** |  |  |  |  |  |  |  |  |  |  |
| I(Place/10) | -0.08** | -0.02 | 0.01 | -0.02 | 0.01 | 0.05 | 0.08** | 0.09** | 0.03 | -0.05 | -0.03 | -0.13** | 0.02 | 0.05 | 0.07** |  |  |  |  |  |  |  |  |  |  |
| Sex: Male | -0.08 | -0.06 | -0.08 | -0.16 | 0.05 | 0.1 | -0.05 | -0.13 | -0.43 | 0.11 | -0.3 | 0.49 | 0.24 | 0.13 | -0.03 |  |  |  |  |  |  |  |  |  |  |
| Age | 0 | 0 | 0 | 0.01 | 0.01 | -0.01 | 0 | 0.03* | 0 | -0.01 | 0 | -0.03* | -0.01 | 0 | 0.01 |  |  |  |  |  |  |  |  |  |  |
| **HR2016** |  |  |  |  |  |  |  |  |  |  |  |  |  |  |  |  |  |  |  |  |  |  |  |  |  |
| (Intercept) | 8.67** | 5.63** | 7.61** | 6.62** | 1.8** | 3.71** | 9.45** | 6.23** | 13.14** | 3.26** | 11.23** | 5.02** | 8.19** | 9.43** |  |  |  |  |  |  |  |  |  |  |  |
| I(Place/10) | -0.15** | -0.05** | -0.06* | -0.01 | 0.01 | -0.01 | 0.07 | 0.08* | 0 | -0.04 | -0.11** | -0.03 | 0.1** | 0.21** |  |  |  |  |  |  |  |  |  |  |  |
| Sex: Male | -0.24 | -0.14 | 0.27 | 0.6* | 0 | 0.01 | 0.24 | 0.31 | 0.36 | -0.09 | -0.64* | -0.12 | -0.32 | -0.23 |  |  |  |  |  |  |  |  |  |  |  |
| Age | 0.01 | 0.01* | 0.01 | -0.01 | 0.01* | 0.01 | -0.01 | -0.01 | -0.02 | 0 | 0.01 | 0 | -0.02* | 0.01 |  |  |  |  |  |  |  |  |  |  |  |
| **HR2017** |  |  |  |  |  |  |  |  |  |  |  |  |  |  |  |  |  |  |  |  |  |  |  |  |  |
| (Intercept) | 7.1** | 5.84** | 2.96** | 6.62** | 2.94** | 9.61** | 6.59** | 5.06** | 7.85** | 5.21** | 5.25** | 13.52** | 10** | 7.25** | 4.19** |  |  |  |  |  |  |  |  |  |  |
| I(Place/10) | -0.12** | -0.04* | -0.01 | -0.04* | 0 | 0.03 | 0.06* | 0.1** | -0.03 | -0.03 | -0.07* | -0.06 | 0.03 | 0.08** | 0.1** |  |  |  |  |  |  |  |  |  |  |
| Sex: Male | 0.01 | 0.01 | 0.06 | 0.11 | 0.05 | 0.36 | 0.04 | 0.01 | 0.04 | -0.01 | -0.72** | -0.04 | -0.27 | 0.18 | 0.23 |  |  |  |  |  |  |  |  |  |  |
| Age | 0.01 | 0.01 | 0 | 0.01 | 0 | -0.01 | 0 | 0.01 | 0.01 | -0.01 | 0 | -0.01 | -0.02 | 0 | 0 |  |  |  |  |  |  |  |  |  |  |
| **HR2018** |  |  |  |  |  |  |  |  |  |  |  |  |  |  |  |  |  |  |  |  |  |  |  |  |  |
| (Intercept) | 9.26** | 6.21** | 8.69** | 7.29** | 2.27** | 3.92** | 8.65** | 5.64** | 12.29** | 2.83** | 11.07** | 4.52** | 7.22** | 10.13** |  |  |  |  |  |  |  |  |  |  |  |
| I(Place/10) | -0.18** | -0.03 | -0.04 | 0 | 0.02 | 0.01 | 0.04 | 0.05 | -0.04 | -0.03 | -0.08* | -0.04* | 0.12** | 0.21** |  |  |  |  |  |  |  |  |  |  |  |
| Sex: Male | -0.23 | -0.25 | -0.12 | 0.12 | -0.16 | -0.02 | 0.2 | 0.22 | 0.09 | 0.09 | -0.04 | -0.05 | 0.21 | -0.08 |  |  |  |  |  |  |  |  |  |  |  |
| Age | 0.01 | 0 | 0 | -0.01 | 0 | 0.01 | 0.01 | 0.01 | 0 | 0 | -0.01 | 0.01 | -0.01 | -0.01 |  |  |  |  |  |  |  |  |  |  |  |
| **HR2021** |  |  |  |  |  |  |  |  |  |  |  |  |  |  |  |  |  |  |  |  |  |  |  |  |  |
| (Intercept) | 7.28** | 6.19** | 3.16** | 7.14** | 3.03** | 9.78** | 7.4** | 5.17** | 8.1** | 4.87** | 4.13** | 12.24** | 9.99** | 7.52** | 4** |  |  |  |  |  |  |  |  |  |  |
| I(Place/10) | -0.11** | -0.03 | -0.01 | -0.07** | -0.01 | 0.02 | 0.04 | 0.05 | -0.03 | -0.05* | -0.04* | -0.02 | 0.09 | 0.08* | 0.1** |  |  |  |  |  |  |  |  |  |  |
| Sex: Male | 0 | -0.13 | -0.07 | -0.17 | 0.05 | 0.21 | 0.24 | 0.21 | 0.32 | -0.07 | -0.03 | 0.02 | -0.14 | -0.32 | -0.11 |  |  |  |  |  |  |  |  |  |  |
| Age | 0 | 0 | 0 | 0.01 | 0 | 0 | -0.02 | 0.02 | 0 | 0 | 0 | 0 | -0.02 | 0 | 0.01 |  |  |  |  |  |  |  |  |  |  |
| **HR2022** |  |  |  |  |  |  |  |  |  |  |  |  |  |  |  |  |  |  |  |  |  |  |  |  |  |
| (Intercept) | 9.01** | 5.66** | 7.91** | 6.49** | 1.96** | 3.24** | 8.9** | 5.91** | 13.94** | 3.75** | 10.55** | 6.54** | 7.05** | 9.28** |  |  |  |  |  |  |  |  |  |  |  |
| I(Place/10) | -0.14** | -0.04* | -0.07** | -0.04 | 0 | 0 | 0.01 | 0.05 | -0.03 | -0.06 | -0.05 | -0.02 | 0.1** | 0.28** |  |  |  |  |  |  |  |  |  |  |  |
| Sex: Male | -0.06 | -0.05 | 0.17 | 0.41* | -0.02 | 0.05 | 0.21 | 0.06 | -0.53 | 0.03 | -0.1 | -0.05 | 0.01 | -0.07 |  |  |  |  |  |  |  |  |  |  |  |
| Age | 0 | 0.01 | 0.01 | 0 | 0.01 | 0.01 | 0 | 0 | -0.01 | -0.01 | 0 | -0.01 | -0.01 | -0.01 |  |  |  |  |  |  |  |  |  |  |  |
| **HURT2015** |  |  |  |  |  |  |  |  |  |  |  |  |  |  |  |  |  |  |  |  |  |  |  |  |  |
| (Intercept) | 5.36** | 4.1** | 5.59** | 5.87** | 4.91** | 6.38** | 7.28** | 4.83** | 6.76** | 7.79** | 6.37** | 8.94** | 9.51** | 7.48** | 9.43** |  |  |  |  |  |  |  |  |  |  |
| I(Place/10) | -0.13* | -0.08 | -0.07 | 0.01 | 0.02 | 0.01 | 0.28** | 0.19** | 0.25** | 0.31** | 0.04 | -0.29 | -0.27** | -0.16 | -0.11 |  |  |  |  |  |  |  |  |  |  |
| Sex: Male | -0.18 | -0.15 | 0.01 | 0.12 | 0.07 | -0.07 | 0.45 | 0.28 | 0.31 | 0.76* | 0.42 | 0.14 | -0.38 | -0.52 | -1.27* |  |  |  |  |  |  |  |  |  |  |
| Age | 0.02 | 0.01 | 0.01 | 0.01 | 0 | 0 | -0.03 | 0 | 0 | -0.02 | -0.01 | 0 | 0 | -0.01 | 0 |  |  |  |  |  |  |  |  |  |  |
| **HURT2016** |  |  |  |  |  |  |  |  |  |  |  |  |  |  |  |  |  |  |  |  |  |  |  |  |  |
| (Intercept) | 5.61** | 2.53** | 4.98** | 5.56** | 4.57** | 6.23** | 6.34** | 5.02** | 7.64** | 7.77** | 6.95** | 8.87** | 9.97** | 7.5** | 9.14** |  |  |  |  |  |  |  |  |  |  |
| I(Place/10) | -0.28** | 0.05 | -0.08 | -0.07 | 0.04 | -0.05 | 0.18* | 0.14** | 0.19** | 0.39** | 0.18 | -0.26* | -0.29** | -0.2* | -0.11 |  |  |  |  |  |  |  |  |  |  |
| Sex: Male | 0.27 | -0.22 | 0 | 0.05 | 0.1 | -0.08 | 0.03 | -0.23 | -0.29 | 0.56 | -0.03 | -0.35 | -0.18 | 0.11 | 0.04 |  |  |  |  |  |  |  |  |  |  |
| Age | 0.01 | 0.03* | 0.02 | 0.03 | 0 | 0.01 | 0.02 | 0.01 | -0.01 | -0.01 | -0.02 | 0.01 | -0.01 | -0.02 | -0.02 |  |  |  |  |  |  |  |  |  |  |
| **HURT2017** |  |  |  |  |  |  |  |  |  |  |  |  |  |  |  |  |  |  |  |  |  |  |  |  |  |
| (Intercept) | 5.58** | 4.14** | 5.77** | 5.79** | 4.42** | 6.52** | 5.86** | 5.25** | 7.13** | 8.7** | 6.76** | 9.32** | 9.68** | 7.16** | 7.92** |  |  |  |  |  |  |  |  |  |  |
| I(Place/10) | 0.03 | 0.01 | 0.02 | 0.06 | 0.06 | 0.04 | 0.21** | 0.13* | 0.19* | 0.15 | 0.07 | -0.23* | -0.32** | -0.28** | -0.12 |  |  |  |  |  |  |  |  |  |  |
| Sex: Male | -0.03 | -0.07 | 0.02 | 0.1 | 0.3* | 0.44 | 0.45 | 0.11 | 0.25 | 0.16 | 0.03 | -0.35 | -0.4 | -0.23 | -0.77 |  |  |  |  |  |  |  |  |  |  |
| Age | 0 | 0 | 0 | 0.01 | 0 | -0.01 | 0.01 | 0 | 0 | -0.01 | -0.01 | -0.01 | 0 | 0 | 0.02 |  |  |  |  |  |  |  |  |  |  |
| **HURT2018** |  |  |  |  |  |  |  |  |  |  |  |  |  |  |  |  |  |  |  |  |  |  |  |  |  |
| (Intercept) | 6.1** | 2.89** | 5.31** | 5.87** | 4.31** | 5.77** | 7.61** | 5.13** | 7.18** | 8.86** | 8.46** | 9.4** | 9.59** | 6.78** | 8.24** |  |  |  |  |  |  |  |  |  |  |
| I(Place/10) | -0.12** | 0.1** | 0.01 | 0 | 0.05 | 0.04 | -0.1 | 0.12** | 0.09* | 0.07 | -0.07 | -0.24** | -0.17** | -0.06 | 0.04 |  |  |  |  |  |  |  |  |  |  |
| Sex: Male | -0.07 | -0.08 | -0.08 | 0.03 | 0.07 | -0.08 | 0.33 | -0.05 | -0.11 | -0.04 | 0.02 | 0.1 | -0.06 | 0.19 | 0.03 |  |  |  |  |  |  |  |  |  |  |
| Age | 0.01 | 0.02* | 0.01 | 0.01 | 0.01 | 0.01 | 0.01 | 0.01 | 0.01 | -0.01 | -0.04 | -0.01 | -0.01 | -0.01 | -0.02 |  |  |  |  |  |  |  |  |  |  |
| **HURT2019** |  |  |  |  |  |  |  |  |  |  |  |  |  |  |  |  |  |  |  |  |  |  |  |  |  |
| (Intercept) | 5.93** | 4.45** | 5.71** | 6.11** | 4.76** | 6.39** | 6.75** | 5.35** | 7.03** | 9.15** | 6.76** | 8.4** | 9.72** | 6.36** | 7.2** |  |  |  |  |  |  |  |  |  |  |
| I(Place/10) | -0.06 | 0 | -0.02 | 0.02 | 0 | 0.02 | 0.07 | 0.09** | 0.1** | 0.22** | 0.01 | -0.11 | -0.25* | -0.15** | 0.08 |  |  |  |  |  |  |  |  |  |  |
| Sex: Male | -0.02 | 0.03 | -0.04 | 0.13 | 0.1 | 0.03 | 0.27 | 0.08 | -0.19 | 0.16 | -0.25 | -0.61 | 0.14 | 0.1 | 0.23 |  |  |  |  |  |  |  |  |  |  |
| Age | 0 | 0 | 0 | 0 | 0 | 0 | 0.01 | 0 | 0.01 | -0.03 | 0 | 0.01 | -0.01 | 0.01 | 0 |  |  |  |  |  |  |  |  |  |  |
| **HURT2020** |  |  |  |  |  |  |  |  |  |  |  |  |  |  |  |  |  |  |  |  |  |  |  |  |  |
| (Intercept) | 5.93** | 4.64** | 5.91** | 6.56** | 4.83** | 6.23** | 7.26** | 5.23** | 6.99** | 7.86** | 6.07** | 8.33** | 9.84** | 6.68** | 7.64** |  |  |  |  |  |  |  |  |  |  |
| I(Place/10) | -0.13 | -0.04 | -0.05 | 0.04 | 0.03 | 0.01 | 0.1 | 0.07 | 0.1 | 0.11 | 0.07 | -0.1 | -0.2* | -0.06 | 0.07 |  |  |  |  |  |  |  |  |  |  |
| Sex: Male | -0.17 | -0.14 | -0.1 | 0.14 | 0.04 | 0.02 | 0.02 | -0.06 | -0.12 | -0.01 | 0.02 | 0.17 | 0.09 | 0.03 | 0.08 |  |  |  |  |  |  |  |  |  |  |
| Age | 0.01 | 0 | 0 | -0.01 | 0 | 0 | 0 | 0.01 | 0.01 | 0.01 | 0 | 0 | -0.02 | -0.01 | -0.01 |  |  |  |  |  |  |  |  |  |  |
| **HURT2022** |  |  |  |  |  |  |  |  |  |  |  |  |  |  |  |  |  |  |  |  |  |  |  |  |  |
| (Intercept) | 5.31** | 4.29** | 5.96** | 6.86** | 5.06** | 6.26** | 7.7** | 5.77** | 7.22** | 8.36** | 6.16** | 7.9** | 8.44** | 6.8** | 7.92** |  |  |  |  |  |  |  |  |  |  |
| I(Place/10) | 0.08 | -0.01 | -0.02 | -0.05 | -0.03 | 0.01 | 0.05 | 0.1 | 0.18* | 0.02 | -0.02 | -0.21 | -0.17 | -0.09 | 0.15 |  |  |  |  |  |  |  |  |  |  |
| Sex: Male | 0.02 | -0.1 | -0.19 | -0.12 | 0.13 | 0.01 | -0.17 | 0 | -0.09 | 0.07 | 0.07 | 0.17 | 0.03 | -0.04 | 0.16 |  |  |  |  |  |  |  |  |  |  |
| Age | 0.01 | 0 | 0 | 0 | 0 | 0 | 0 | -0.01 | 0 | 0 | 0 | 0.01 | 0.01 | -0.01 | -0.01 |  |  |  |  |  |  |  |  |  |  |
| **RR2012** |  |  |  |  |  |  |  |  |  |  |  |  |  |  |  |  |  |  |  |  |  |  |  |  |  |
| (Intercept) | 16.84** | 17.73** | 19.7** | 21.22** | 24.84** |  |  |  |  |  |  |  |  |  |  |  |  |  |  |  |  |  |  |  |  |
| I(Place/10) | -0.1** | -0.04* | 0 | 0.1** | 0.02 |  |  |  |  |  |  |  |  |  |  |  |  |  |  |  |  |  |  |  |  |
| Sex: Male | -0.77* | -0.29 | -0.11 | -0.32 | 1.59* |  |  |  |  |  |  |  |  |  |  |  |  |  |  |  |  |  |  |  |  |
| Age | 0.01 | 0 | 0 | 0.02 | -0.03 |  |  |  |  |  |  |  |  |  |  |  |  |  |  |  |  |  |  |  |  |
| **RR2013** |  |  |  |  |  |  |  |  |  |  |  |  |  |  |  |  |  |  |  |  |  |  |  |  |  |
| (Intercept) | 15.65** | 16.87** | 18.92** | 21.91** | 26.65** |  |  |  |  |  |  |  |  |  |  |  |  |  |  |  |  |  |  |  |  |
| I(Place/10) | -0.09** | -0.02 | 0.01 | 0.08** | 0.02 |  |  |  |  |  |  |  |  |  |  |  |  |  |  |  |  |  |  |  |  |
| Sex: Male | -0.28 | -0.24 | 0.23 | 0.06 | 0.23 |  |  |  |  |  |  |  |  |  |  |  |  |  |  |  |  |  |  |  |  |
| Age | 0.02* | 0.02 | 0.01 | 0 | -0.06* |  |  |  |  |  |  |  |  |  |  |  |  |  |  |  |  |  |  |  |  |
| **RR2014** |  |  |  |  |  |  |  |  |  |  |  |  |  |  |  |  |  |  |  |  |  |  |  |  |  |
| (Intercept) | 16.73** | 17.96** | 19.03** | 21.43** | 25** |  |  |  |  |  |  |  |  |  |  |  |  |  |  |  |  |  |  |  |  |
| I(Place/10) | -0.08** | -0.02 | 0.04** | 0.1** | -0.04 |  |  |  |  |  |  |  |  |  |  |  |  |  |  |  |  |  |  |  |  |
| Sex: Male | -0.44* | 0.09 | 0.27 | 0.12 | 0.01 |  |  |  |  |  |  |  |  |  |  |  |  |  |  |  |  |  |  |  |  |
| Age | 0.01 | -0.01 | 0 | 0 | -0.01 |  |  |  |  |  |  |  |  |  |  |  |  |  |  |  |  |  |  |  |  |
| **RR2015** |  |  |  |  |  |  |  |  |  |  |  |  |  |  |  |  |  |  |  |  |  |  |  |  |  |
| (Intercept) | 16.95** | 17.29** | 18.07** | 21.03** | 26.63** |  |  |  |  |  |  |  |  |  |  |  |  |  |  |  |  |  |  |  |  |
| I(Place/10) | -0.11** | -0.05** | 0.01 | 0.13** | 0.02 |  |  |  |  |  |  |  |  |  |  |  |  |  |  |  |  |  |  |  |  |
| Sex: Male | -0.25 | -0.13 | 0.04 | 0.24 | 0.09 |  |  |  |  |  |  |  |  |  |  |  |  |  |  |  |  |  |  |  |  |
| Age | 0.01 | 0.01 | 0.03* | 0 | -0.05 |  |  |  |  |  |  |  |  |  |  |  |  |  |  |  |  |  |  |  |  |
| **RR2016** |  |  |  |  |  |  |  |  |  |  |  |  |  |  |  |  |  |  |  |  |  |  |  |  |  |
| (Intercept) | 16.59** | 17.6** | 19.51** | 21.5** | 24.79** |  |  |  |  |  |  |  |  |  |  |  |  |  |  |  |  |  |  |  |  |
| I(Place/10) | -0.11** | -0.04** | 0.04** | 0.12** | -0.01 |  |  |  |  |  |  |  |  |  |  |  |  |  |  |  |  |  |  |  |  |
| Sex: Male | -0.55* | -0.2 | -0.31 | 0.16 | 0.89 |  |  |  |  |  |  |  |  |  |  |  |  |  |  |  |  |  |  |  |  |
| Age | 0.02 | 0.01 | -0.01 | 0 | -0.01 |  |  |  |  |  |  |  |  |  |  |  |  |  |  |  |  |  |  |  |  |
| **RR2017** |  |  |  |  |  |  |  |  |  |  |  |  |  |  |  |  |  |  |  |  |  |  |  |  |  |
| (Intercept) | 17.12** | 17.16** | 18.89** | 21.44** | 25.35** |  |  |  |  |  |  |  |  |  |  |  |  |  |  |  |  |  |  |  |  |
| I(Place/10) | -0.11** | -0.05** | 0.03 | 0.13** | 0 |  |  |  |  |  |  |  |  |  |  |  |  |  |  |  |  |  |  |  |  |
| Sex: Male | -0.43 | -0.16 | 0.21 | -0.29 | 0.68 |  |  |  |  |  |  |  |  |  |  |  |  |  |  |  |  |  |  |  |  |
| Age | 0 | 0.01 | 0 | 0 | -0.02 |  |  |  |  |  |  |  |  |  |  |  |  |  |  |  |  |  |  |  |  |
| **RR2018** |  |  |  |  |  |  |  |  |  |  |  |  |  |  |  |  |  |  |  |  |  |  |  |  |  |
| (Intercept) | 20.3** | 22.07** | 25.08** | 32.42** |  |  |  |  |  |  |  |  |  |  |  |  |  |  |  |  |  |  |  |  |  |
| I(Place/10) | -0.1** | 0.02 | 0.16** | -0.08 |  |  |  |  |  |  |  |  |  |  |  |  |  |  |  |  |  |  |  |  |  |
| Sex: Male | -0.42 | 0.36 | 0.22 | -0.17 |  |  |  |  |  |  |  |  |  |  |  |  |  |  |  |  |  |  |  |  |  |
| Age | 0.01 | 0 | 0.01 | -0.01 |  |  |  |  |  |  |  |  |  |  |  |  |  |  |  |  |  |  |  |  |  |
| **RR2019** |  |  |  |  |  |  |  |  |  |  |  |  |  |  |  |  |  |  |  |  |  |  |  |  |  |
| (Intercept) | 20.03** | 21.32** | 24.92** | 33.32** |  |  |  |  |  |  |  |  |  |  |  |  |  |  |  |  |  |  |  |  |  |
| I(Place/10) | -0.09** | -0.02 | 0.16** | -0.06 |  |  |  |  |  |  |  |  |  |  |  |  |  |  |  |  |  |  |  |  |  |
| Sex: Male | -0.66 | -0.21 | 0.15 | 0.78 |  |  |  |  |  |  |  |  |  |  |  |  |  |  |  |  |  |  |  |  |  |
| Age | 0.02 | 0.03 | 0.01 | -0.05 |  |  |  |  |  |  |  |  |  |  |  |  |  |  |  |  |  |  |  |  |  |
| **RR2020** |  |  |  |  |  |  |  |  |  |  |  |  |  |  |  |  |  |  |  |  |  |  |  |  |  |
| (Intercept) | 21.02** | 22.85** | 25.42** | 30.77** |  |  |  |  |  |  |  |  |  |  |  |  |  |  |  |  |  |  |  |  |  |
| I(Place/10) | -0.07** | 0 | 0.09** | 0 |  |  |  |  |  |  |  |  |  |  |  |  |  |  |  |  |  |  |  |  |  |
| Sex: Male | -0.51 | -0.09 | -0.04 | 0.64 |  |  |  |  |  |  |  |  |  |  |  |  |  |  |  |  |  |  |  |  |  |
| Age | -0.01 | -0.01 | 0.02 | -0.01 |  |  |  |  |  |  |  |  |  |  |  |  |  |  |  |  |  |  |  |  |  |
| **RR2021** |  |  |  |  |  |  |  |  |  |  |  |  |  |  |  |  |  |  |  |  |  |  |  |  |  |
| (Intercept) | 16.18** | 17.68** | 19.08** | 21.78** | 25.34** |  |  |  |  |  |  |  |  |  |  |  |  |  |  |  |  |  |  |  |  |
| I(Place/10) | -0.11** | -0.05** | 0.02 | 0.1** | 0.04 |  |  |  |  |  |  |  |  |  |  |  |  |  |  |  |  |  |  |  |  |
| Sex: Male | 0.04 | 0.1 | 0.27 | -0.22 | -0.12 |  |  |  |  |  |  |  |  |  |  |  |  |  |  |  |  |  |  |  |  |
| Age | 0.02 | 0.01 | 0 | 0 | -0.03 |  |  |  |  |  |  |  |  |  |  |  |  |  |  |  |  |  |  |  |  |
| **RR2022** |  |  |  |  |  |  |  |  |  |  |  |  |  |  |  |  |  |  |  |  |  |  |  |  |  |
| (Intercept) | 16.25** | 17.63** | 18.76** | 21.96** | 25.44** |  |  |  |  |  |  |  |  |  |  |  |  |  |  |  |  |  |  |  |  |
| I(Place/10) | -0.13** | -0.09** | 0.02 | 0.18** | 0.03 |  |  |  |  |  |  |  |  |  |  |  |  |  |  |  |  |  |  |  |  |
| Sex: Male | -0.51 | -0.24 | -0.16 | 0.11 | 0.82 |  |  |  |  |  |  |  |  |  |  |  |  |  |  |  |  |  |  |  |  |
| Age | 0.02 | 0.02 | 0.01 | -0.02 | -0.04 |  |  |  |  |  |  |  |  |  |  |  |  |  |  |  |  |  |  |  |  |
| **TP2013** |  |  |  |  |  |  |  |  |  |  |  |  |  |  |  |  |  |  |  |  |  |  |  |  |  |
| (Intercept) | 8.43** | 8.25** | 4.31** | 8.29** | 9.19** | 5.13** | 10.26** | 11.33** | 7.4** | 13.21** | 14.29** |  |  |  |  |  |  |  |  |  |  |  |  |  |  |
| I(Place/10) | -0.18** | -0.1** | 0.05 | 0 | 0.05 | 0.01 | 0.11** | 0.17** | 0.04 | -0.05 | -0.07 |  |  |  |  |  |  |  |  |  |  |  |  |  |  |
| Sex: Male | -0.2 | -0.2 | 0.06 | 0.18 | -0.3 | 0.3 | -0.32 | -0.31 | -0.18 | 0.68 | 0.28 |  |  |  |  |  |  |  |  |  |  |  |  |  |  |
| Age | 0 | 0 | 0 | 0.02 | 0.02 | 0.03 | 0 | -0.01 | -0.01 | -0.02 | -0.03 |  |  |  |  |  |  |  |  |  |  |  |  |  |  |
| **TP2014** |  |  |  |  |  |  |  |  |  |  |  |  |  |  |  |  |  |  |  |  |  |  |  |  |  |
| (Intercept) | 17.52** | 14.13** | 12.15** | 15.39** | 23.05** | 17.56** |  |  |  |  |  |  |  |  |  |  |  |  |  |  |  |  |  |  |  |
| I(Place/10) | -0.1** | -0.03 | 0 | 0.12** | -0.02 | 0.03 |  |  |  |  |  |  |  |  |  |  |  |  |  |  |  |  |  |  |  |
| Sex: Male | -0.58 | 0.28 | 0.33 | -0.08 | -0.13 | 0.23 |  |  |  |  |  |  |  |  |  |  |  |  |  |  |  |  |  |  |  |
| Age | -0.01 | -0.01 | -0.01 | 0.03* | 0.03 | -0.02 |  |  |  |  |  |  |  |  |  |  |  |  |  |  |  |  |  |  |  |
| **TP2015** |  |  |  |  |  |  |  |  |  |  |  |  |  |  |  |  |  |  |  |  |  |  |  |  |  |
| (Intercept) | 24.41** | 6.69** | 11.84** | 6.66** | 13.21** | 26.94** | 10.3** |  |  |  |  |  |  |  |  |  |  |  |  |  |  |  |  |  |  |
| I(Place/10) | -0.12** | -0.01 | -0.04 | 0.08** | 0.07** | -0.02 | 0.04 |  |  |  |  |  |  |  |  |  |  |  |  |  |  |  |  |  |  |
| Sex: Male | -0.84 | 0.27 | -0.44 | -0.08 | 0.23 | 0.37 | 0.45 |  |  |  |  |  |  |  |  |  |  |  |  |  |  |  |  |  |  |
| Age | 0.01 | 0 | 0.02 | 0.02* | 0.02 | -0.05** | -0.02 |  |  |  |  |  |  |  |  |  |  |  |  |  |  |  |  |  |  |
| **TP2016** |  |  |  |  |  |  |  |  |  |  |  |  |  |  |  |  |  |  |  |  |  |  |  |  |  |
| (Intercept) | 23.18** | 17.79** | 7.52** | 13.51** | 26.87** | 11.13** |  |  |  |  |  |  |  |  |  |  |  |  |  |  |  |  |  |  |  |
| I(Place/10) | -0.1** | -0.04* | 0.05** | 0.08** | -0.01 | 0.01 |  |  |  |  |  |  |  |  |  |  |  |  |  |  |  |  |  |  |  |
| Sex: Male | -0.19 | 0.12 | -0.35 | -0.16 | 0.3 | 0.32 |  |  |  |  |  |  |  |  |  |  |  |  |  |  |  |  |  |  |  |
| Age | 0.03 | 0.04** | 0.01 | 0.01 | -0.05* | -0.04* |  |  |  |  |  |  |  |  |  |  |  |  |  |  |  |  |  |  |  |
| **TP2017** |  |  |  |  |  |  |  |  |  |  |  |  |  |  |  |  |  |  |  |  |  |  |  |  |  |
| (Intercept) | 22.78** | 17.72** | 7.06** | 14.11** | 26.67** | 11.43** |  |  |  |  |  |  |  |  |  |  |  |  |  |  |  |  |  |  |  |
| I(Place/10) | -0.08** | 0.03 | 0.06** | 0.04* | -0.04 | 0 |  |  |  |  |  |  |  |  |  |  |  |  |  |  |  |  |  |  |  |
| Sex: Male | -0.85* | 0.02 | 0.27 | -0.05 | 0.94 | -0.1 |  |  |  |  |  |  |  |  |  |  |  |  |  |  |  |  |  |  |  |
| Age | 0.05* | 0.03* | 0.01 | 0.01 | -0.06* | -0.03* |  |  |  |  |  |  |  |  |  |  |  |  |  |  |  |  |  |  |  |
| **TP2018** |  |  |  |  |  |  |  |  |  |  |  |  |  |  |  |  |  |  |  |  |  |  |  |  |  |
| (Intercept) | 8.92** | 20.92** | 12.36** | 21.3** | 18.81** | 17.67** |  |  |  |  |  |  |  |  |  |  |  |  |  |  |  |  |  |  |  |
| I(Place/10) | -0.06** | -0.01 | -0.05** | 0.08** | -0.05* | 0.1** |  |  |  |  |  |  |  |  |  |  |  |  |  |  |  |  |  |  |  |
| Sex: Male | -0.34 | -0.12 | 0.03 | 0.1 | 0.22 | 0.08 |  |  |  |  |  |  |  |  |  |  |  |  |  |  |  |  |  |  |  |
| Age | 0.01 | 0.04* | 0.02* | 0.01 | -0.03 | -0.05* |  |  |  |  |  |  |  |  |  |  |  |  |  |  |  |  |  |  |  |
| **TP2019** |  |  |  |  |  |  |  |  |  |  |  |  |  |  |  |  |  |  |  |  |  |  |  |  |  |
| (Intercept) | 9.76** | 14.85** | 6.65** | 12.14** | 19.96** | 18.02** | 13.47** | 5.18** |  |  |  |  |  |  |  |  |  |  |  |  |  |  |  |  |  |
| I(Place/10) | -0.08** | -0.04** | -0.01 | -0.01 | 0.11** | 0.03 | -0.02 | 0.02** |  |  |  |  |  |  |  |  |  |  |  |  |  |  |  |  |  |
| Sex: Male | -0.21 | -0.36 | -0.03 | 0.04 | 0.35 | 0.14 | -0.02 | 0.06 |  |  |  |  |  |  |  |  |  |  |  |  |  |  |  |  |  |
| Age | 0 | 0.02 | 0.01 | 0.01 | 0.02 | -0.01 | -0.04** | -0.01 |  |  |  |  |  |  |  |  |  |  |  |  |  |  |  |  |  |
| **TP2020** |  |  |  |  |  |  |  |  |  |  |  |  |  |  |  |  |  |  |  |  |  |  |  |  |  |
| (Intercept) | 9** | 17.06** | 19.63** | 6.98** | 13.47** | 17.59** | 11.69** | 4.63** |  |  |  |  |  |  |  |  |  |  |  |  |  |  |  |  |  |
| I(Place/10) | -0.09** | -0.07** | -0.03 | 0.04** | 0.06** | 0.08** | 0 | 0.01 |  |  |  |  |  |  |  |  |  |  |  |  |  |  |  |  |  |
| Sex: Male | -0.32 | -0.27 | 0.04 | 0.07 | 0.04 | -0.12 | 0.27 | 0.29 |  |  |  |  |  |  |  |  |  |  |  |  |  |  |  |  |  |
| Age | 0 | 0.01 | 0.01 | 0.01 | -0.01 | 0 | -0.01 | -0.01 |  |  |  |  |  |  |  |  |  |  |  |  |  |  |  |  |  |
| **TP2021** |  |  |  |  |  |  |  |  |  |  |  |  |  |  |  |  |  |  |  |  |  |  |  |  |  |
| (Intercept) | 18.31** | 14.1** | 5.61** | 6.29** | 7.05** | 8.31** | 12.33** | 9.86** | 7.02** | 5.76** | 5.38** |  |  |  |  |  |  |  |  |  |  |  |  |  |  |
| I(Place/10) | -0.12** | -0.03 | -0.01 | -0.01 | 0.05** | 0.03** | 0.11** | 0.04 | -0.04** | -0.01 | 0 |  |  |  |  |  |  |  |  |  |  |  |  |  |  |
| Sex: Male | -0.24 | -0.25 | -0.03 | 0.05 | 0.06 | 0.11 | -0.11 | 0 | 0.1 | -0.03 | 0.32 |  |  |  |  |  |  |  |  |  |  |  |  |  |  |
| Age | -0.01 | 0.02 | 0.01 | 0.01 | 0.01 | 0 | 0 | -0.01 | 0 | -0.01 | 0 |  |  |  |  |  |  |  |  |  |  |  |  |  |  |
| **TP2022** |  |  |  |  |  |  |  |  |  |  |  |  |  |  |  |  |  |  |  |  |  |  |  |  |  |
| (Intercept) | 8.15** | 9.21** | 7.25** | 12.59** | 6.43** | 8.8** | 8** | 5.06** | 8.16** | 9.72** | 11.55** | 5.04** |  |  |  |  |  |  |  |  |  |  |  |  |  |
| I(Place/10) | -0.06** | -0.04** | -0.01 | -0.02 | -0.01 | 0.03** | 0.03** | 0.01 | 0.02 | -0.02 | 0 | 0.05** |  |  |  |  |  |  |  |  |  |  |  |  |  |
| Sex: Male | -0.25 | -0.31* | -0.06 | 0.12 | 0.1 | 0.08 | 0.15 | 0.07 | -0.14 | -0.21 | 0.22 | 0.27 |  |  |  |  |  |  |  |  |  |  |  |  |  |
| Age | 0 | 0.01 | 0 | 0.01 | 0 | 0 | 0.01 | 0 | -0.01 | 0 | -0.01 | -0.01 |  |  |  |  |  |  |  |  |  |  |  |  |  |
| **UTMB2013** |  |  |  |  |  |  |  |  |  |  |  |  |  |  |  |  |  |  |  |  |  |  |  |  |  |
| (Intercept) | 5.62** | 2.45** | 4.76** | 4.04** | 4.25** | 2.16** | 6.37** | 1.9** | 3.22** | 2.06** | 2.24** | 5.4** | 3.89** | 2.81** | 4.29** | 4.51** | 7.53** | 6.69** | 3.89** | 5.11** | 3.2** | 7.58** | 2.19** | 3.85** |  |
| I(Place/10) | -0.01** | 0** | 0 | 0** | 0** | 0** | 0** | 0** | 0** | 0** | 0** | 0* | 0 | 0 | 0 | 0** | 0.01** | 0.01** | 0** | 0** | 0** | 0** | 0** | 0* |  |
| Sex: Male | -0.12** | -0.07* | 0.11 | 0.03 | -0.02 | -0.04 | 0.1 | -0.02 | 0.08* | -0.03 | -0.05 | 0.17 | 0 | 0.04 | -0.02 | -0.16** | -0.06 | 0.11 | -0.05 | 0.13 | -0.05 | -0.04 | -0.02 | -0.02 |  |
| **UTMB2014** |  |  |  |  |  |  |  |  |  |  |  |  |  |  |  |  |  |  |  |  |  |  |  |  |  |
| (Intercept) | 5.49** | 2.53** | 4.47** | 4.35** | 4.37** | 2.22** | 6.38** | 1.84** | 3.25** | 1.97** | 2.06** | 5.24** | 3.77** | 2.74** | 4.43** | 4.28** | 7.48** | 8.06** | 2.4** | 5.27** | 3.29** | 7.87** | 2.7** | 3.58** |  |
| I(Place/10) | -0.01** | 0* | 0** | 0** | 0** | 0** | 0** | 0** | 0** | 0** | 0 | 0 | 0** | 0** | 0 | 0** | 0.01** | 0.01** | 0.01** | 0** | 0* | -0.01** | 0** | 0 |  |
| Sex: Male | -0.06 | -0.11* | -0.02 | 0.16* | -0.06 | -0.1** | 0.12 | 0.02 | 0.02 | -0.03 | 0.01 | 0.18* | -0.04 | 0 | -0.12 | -0.08 | -0.1 | 0.24 | -0.05 | 0.01 | 0.01 | 0.06 | -0.01 | -0.04 |  |
| **UTMB2015** |  |  |  |  |  |  |  |  |  |  |  |  |  |  |  |  |  |  |  |  |  |  |  |  |  |
| (Intercept) | 5.34** | 2.37** | 4.3** | 4.17** | 4.16** | 2.06** | 6.27** | 4.21** | 3.16** | 1.84** | 1.91** | 5.11** | 3.64** | 2.74** | 4.41** | 4.26** | 7.57** | 8.57** | 2.4** | 5.27** | 3.15** | 7.45** | 2.43** | 3.2** |  |
| I(Place/10) | 0** | 0* | 0** | 0* | 0** | 0 | 0** | 0** | 0** | 0** | 0 | 0** | 0** | 0** | 0** | 0** | 0.01** | 0.01** | 0** | 0 | 0** | -0.01** | 0** | 0** |  |
| Sex: Male | -0.05 | -0.04 | 0.05 | 0.2** | -0.02 | -0.04 | 0.09 | 0.03 | 0.03 | -0.05* | -0.01 | 0.21** | -0.01 | 0 | -0.09 | -0.14** | -0.09 | 0.02 | -0.05 | 0.08 | 0 | -0.04 | -0.04 | -0.05 |  |
| **UTMB2016** |  |  |  |  |  |  |  |  |  |  |  |  |  |  |  |  |  |  |  |  |  |  |  |  |  |
| (Intercept) | 5.29** | 2.34** | 4.29** | 4.26** | 4.15** | 2.03** | 6.17** | 4.03** | 3.09** | 1.84** | 1.93** | 5** | 3.81** | 2.75** | 4.34** | 4.3** | 7.18** | 8.49** | 2.73** | 5.35** | 3.18** | 7.6** | 2.55** | 3.25** |  |
| I(Place/10) | 0** | 0* | 0** | 0** | 0** | 0 | 0 | 0 | 0** | 0** | 0 | 0** | 0** | 0 | 0 | 0** | 0.01** | 0.01** | 0** | 0 | 0** | -0.01** | 0** | 0* |  |
| Sex: Male | -0.03 | -0.03 | 0.07 | 0.14* | -0.05 | -0.04 | 0.11 | 0.01 | 0.05 | -0.02 | -0.02 | 0.28** | -0.01 | 0.05 | -0.01 | -0.14** | 0.04 | 0.12 | -0.01 | 0.06 | -0.1 | -0.23 | -0.15** | -0.05 |  |
| **UTMB2017** |  |  |  |  |  |  |  |  |  |  |  |  |  |  |  |  |  |  |  |  |  |  |  |  |  |
| (Intercept) | 5.43** | 2.56** | 4.36** | 4.4** | 4.43** | 2.25** | 6.89** | 1.8** | 3.22** | 1.94** | 1.97** | 5.34** | 3.83** | 2.84** | 4.73** | 4.17** | 6.82** | 8.51** | 2.68** | 4.09** | 4.47** | 3.25** | 6.41** | 3.62** |  |
| I(Place/10) | -0.01** | 0** | 0** | 0** | 0** | 0 | 0** | 0* | 0 | 0** | 0** | 0 | 0 | 0** | 0* | 0** | 0.01** | 0.01** | 0.01** | 0** | 0** | 0** | -0.01** | 0 |  |
| Sex: Male | -0.07 | -0.06* | -0.01 | 0.09 | -0.08* | -0.11** | -0.03 | -0.03 | 0.01 | -0.06** | -0.04 | 0.19* | -0.06 | -0.04 | -0.02 | -0.12** | -0.01 | 0.2 | 0 | 0.13 | 0.03 | 0.19* | -0.08 | 0.01 |  |
| **UTMB2018** |  |  |  |  |  |  |  |  |  |  |  |  |  |  |  |  |  |  |  |  |  |  |  |  |  |
| (Intercept) | 5.42** | 2.52** | 4.4** | 4.44** | 4.37** | 2.1** | 6.85** | 1.82** | 3.21** | 1.95** | 2.01** | 5.54** | 3.85** | 2.79** | 4.47** | 1.53** | 2.84** | 7.21** | 8.43** | 2.61** | 4.46** | 4.27** | 3.36** | 6.12** | 3.44** |
| I(Place/10) | 0** | 0** | 0** | 0** | 0** | 0** | 0** | 0 | 0 | 0** | 0 | 0** | 0 | 0 | 0 | 0** | 0** | 0.01** | 0.01** | 0** | 0 | 0** | 0** | -0.01** | 0 |
| Sex: Male | -0.07 | -0.08** | -0.01 | 0.07 | -0.06 | -0.09** | 0 | -0.02 | 0.01 | -0.05* | -0.06* | 0.16 | -0.05 | -0.01 | -0.01 | -0.02 | -0.11** | 0.04 | 0.18 | 0 | 0.06 | 0.1 | 0.08 | -0.06 | -0.03 |
| **UTMB2019** |  |  |  |  |  |  |  |  |  |  |  |  |  |  |  |  |  |  |  |  |  |  |  |  |  |
| (Intercept) | 2.26** | 5.51** | 4.36** | 4.34** | 4.21** | 1.9** | 6.15** | 4.44** | 3.1** | 1.83** | 1.89** | 5.22** | 3.68** | 2.64** | 4.37** | 4.21** | 7.35** | 8.18** | 2.51** | 4.08** | 4.26** | 7.49** | 2.63** | 3.37** |  |
| I(Place/10) | 0** | 0** | 0** | 0 | 0** | 0 | 0** | 0** | 0** | 0** | 0* | 0** | 0** | 0 | 0 | 0** | 0.01** | 0.01** | 0** | 0* | 0** | -0.01** | 0** | 0 |  |
| Sex: Male | -0.02 | -0.23** | -0.01 | 0.15** | -0.08* | -0.07** | 0.05 | 0.04 | 0.08* | -0.03 | -0.06* | 0.22** | 0.03 | -0.01 | 0.03 | -0.1 | -0.05 | 0.22* | -0.01 | 0 | -0.04 | 0.03 | -0.06 | -0.08 |  |
| **UTMB2021** |  |  |  |  |  |  |  |  |  |  |  |  |  |  |  |  |  |  |  |  |  |  |  |  |  |
| (Intercept) | 5.27** | 2.23** | 4.18** | 4.29** | 4.21** | 1.76** | 6.57** | 4.08** | 3.27** | 1.91** | 2.05** | 5.51** | 6.34** | 4.51** | 4.37** | 7.1** | 8.21** | 2.43** | 4.06** | 4.12** | 7.46** | 2.62** | 3.44** |  |  |
| I(Place/10) | -0.01** | 0 | 0** | 0 | 0** | 0** | 0 | 0 | 0** | 0** | 0* | 0 | 0 | 0 | 0** | 0.01** | 0.01** | 0.01** | 0** | 0* | -0.01** | 0** | 0 |  |  |
| Sex: Male | -0.09 | -0.01 | 0.04 | 0.16** | -0.01 | -0.01 | 0.08 | -0.02 | 0.02 | -0.05* | -0.05 | 0.22* | -0.04 | -0.03 | -0.11* | -0.05 | 0.18 | -0.04 | -0.04 | -0.01 | -0.06 | -0.03 | -0.07 |  |  |
| **UTMB2022** |  |  |  |  |  |  |  |  |  |  |  |  |  |  |  |  |  |  |  |  |  |  |  |  |  |
| (Intercept) | 5.16** | 2.75** | 4.11** | 4.37** | 4.26** | 1.82** | 6.49** | 4.01** | 3.17** | 1.9** | 1.96** | 5.3** | 6.44** | 4.54** | 4.5** | 7.25** | 8.22** | 2.47** | 3.84** | 4.2** | 7.35** | 2.52** | 3.39** |  |  |
| I(Place/10) | 0** | 0** | 0** | 0** | 0** | 0* | 0** | 0 | 0** | 0** | 0 | 0** | 0** | 0** | 0** | 0** | 0.01** | 0** | 0** | 0** | 0** | 0** | 0* |  |  |
| Sex: Male | -0.08 | -0.07* | -0.03 | 0.13** | -0.04 | -0.04 | 0.07 | 0 | 0.06 | -0.04 | -0.06* | 0.19* | -0.02 | 0.04 | -0.08 | -0.13 | 0.14 | 0 | 0.18 | -0.01 | 0 | -0.03 | -0.15 |  |  |
| **WS2012** |  |  |  |  |  |  |  |  |  |  |  |  |  |  |  |  |  |  |  |  |  |  |  |  |  |
| (Intercept) | 9.5** | 4.93** | 6.22** | 6.25** | 3.99** | 2.75** | 4.07** | 5.31** | 4.42** | 3.99** | 6.35** | 7.7** | 7.55** | 2.52** | 6.42** | 5.73** | 4.78** | 4.13** | 2.86** | 1.35** |  |  |  |  |  |
| I(Place/10) | -0.05** | -0.02** | -0.02** | -0.02* | -0.01 | 0 | 0 | -0.01** | 0.02** | -0.01** | 0.01* | 0.05** | 0.07** | 0 | 0.03** | 0.01 | -0.02** | -0.02** | -0.02** | -0.01** |  |  |  |  |  |
| Sex: Male | -0.08 | -0.14 | -0.13 | -0.1 | 0.13 | 0.11 | -0.01 | -0.11 | 0.03 | 0.02 | 0.12 | -0.03 | 0.1 | -0.04 | -0.09 | -0.11 | 0.03 | 0.19 | 0.09 | 0.07 |  |  |  |  |  |
| Age | 0.01 | 0.01* | 0.01** | 0.01 | 0.01 | 0 | 0 | 0 | 0 | 0 | -0.01 | 0 | -0.02** | 0.01 | -0.02* | -0.01 | 0 | -0.01 | 0 | 0 |  |  |  |  |  |
| **WS2013** |  |  |  |  |  |  |  |  |  |  |  |  |  |  |  |  |  |  |  |  |  |  |  |  |  |
| (Intercept) | 9.34** | 5.22** | 5.93** | 5.88** | 4.07** | 2.98** | 3.77** | 5.65** | 4.74** | 3.88** | 6.15** | 7.98** | 7.29** | 2.7** | 6.21** | 5.48** | 4.85** | 6.65** | 1.32** |  |  |  |  |  |  |
| I(Place/10) | -0.05** | -0.02** | -0.01* | -0.02** | -0.01 | 0 | -0.01* | -0.01 | 0.03** | -0.01** | 0.01 | 0.05** | 0.05** | 0.01* | 0.01 | -0.01 | -0.02** | 0 | 0 |  |  |  |  |  |  |
| Sex: Male | -0.29 | -0.16 | -0.24 | -0.06 | 0 | 0.05 | -0.06 | 0.26 | -0.1 | 0.07 | 0.2 | -0.01 | 0.17 | 0.03 | 0.11 | 0.09 | 0.01 | 0 | 0.04 |  |  |  |  |  |  |
| Age | 0.01 | 0 | 0.01* | 0.01* | 0.01 | 0 | 0 | 0 | 0 | 0.01 | 0 | 0 | -0.01 | 0 | -0.01 | -0.01 | -0.01 | -0.01 | 0 |  |  |  |  |  |  |
| **WS2014** |  |  |  |  |  |  |  |  |  |  |  |  |  |  |  |  |  |  |  |  |  |  |  |  |  |
| (Intercept) | 9.65** | 5.23** | 6.44** | 6.22** | 4.26** | 2.64** | 4.05** | 5.56** | 4.47** | 4.17** | 6.32** | 7.74** | 7.08** | 2.74** | 5.93** | 5.13** | 4.51** | 3.97** | 2.69** | 1.3** |  |  |  |  |  |
| I(Place/10) | -0.05** | -0.03** | -0.02** | -0.01* | 0.01** | 0 | 0 | 0 | 0.02** | -0.01** | 0.02** | 0.05** | 0.06** | 0.02** | 0.01 | -0.01 | -0.03** | -0.02** | -0.02** | 0* |  |  |  |  |  |
| Sex: Male | -0.28 | -0.17 | -0.24 | -0.2 | 0.18* | 0.09 | 0.07 | 0.02 | 0.07 | 0.03 | 0.18 | -0.01 | -0.11 | 0.02 | -0.07 | 0.1 | 0.03 | 0.09 | 0.03 | 0.04 |  |  |  |  |  |
| Age | 0.01 | 0.01 | 0.01 | 0.01 | -0.01 | 0 | 0 | 0 | -0.01 | 0 | -0.01* | 0 | -0.01 | 0 | 0 | 0 | 0 | 0 | 0 | 0 |  |  |  |  |  |
| **WS2015** |  |  |  |  |  |  |  |  |  |  |  |  |  |  |  |  |  |  |  |  |  |  |  |  |  |
| (Intercept) | 9.32** | 4.88** | 6.26** | 6.01** | 3.89** | 2.6** | 3.76** | 5.57** | 4.57** | 4.48** | 6.77** | 7.87** | 8.09** | 2.44** | 5.76** | 4.89** | 4.58** | 6.57** | 1.36** |  |  |  |  |  |  |
| I(Place/10) | -0.04** | -0.01 | -0.02** | 0.01 | 0 | 0 | 0.01 | 0.01 | 0.02* | -0.01** | 0.02 | 0.06** | 0.05** | 0.01 | 0.01 | -0.02* | -0.03** | -0.04** | 0 |  |  |  |  |  |  |
| Sex: Male | -0.3* | -0.16 | -0.13 | -0.01 | 0.06 | 0.03 | 0.04 | 0.25 | 0.18 | 0.05 | 0 | -0.09 | 0.12 | 0.03 | 0.05 | 0.07 | 0.02 | -0.12 | -0.01 |  |  |  |  |  |  |
| Age | 0.01 | 0.01 | 0.01 | 0 | 0.01 | 0 | 0 | 0 | 0 | -0.01 | -0.01 | 0 | -0.02 | 0 | 0 | 0.01 | 0 | 0 | 0 |  |  |  |  |  |  |
| **WS2016** |  |  |  |  |  |  |  |  |  |  |  |  |  |  |  |  |  |  |  |  |  |  |  |  |  |
| (Intercept) | 9.96** | 5.16** | 6.23** | 5.99** | 3.88** | 2.81** | 3.69** | 5.5** | 8.73** | 6.51** | 7.92** | 7.2** | 2.62** | 5.76** | 5.11** | 5.08** | 6.83** | 1.32** |  |  |  |  |  |  |  |
| I(Place/10) | -0.06** | -0.03** | -0.01 | -0.01 | 0.01 | -0.02* | 0.01 | 0.01 | 0 | 0.01 | 0.07** | 0.07** | 0 | 0.01 | -0.03** | -0.04** | -0.03** | 0 |  |  |  |  |  |  |  |
| Sex: Male | -0.35** | -0.2** | -0.23* | -0.12 | -0.03 | 0.12 | 0.06 | 0.22 | 0.21 | 0.06 | 0.2 | 0.24 | 0.07 | -0.01 | -0.03 | -0.03 | 0.04 | 0.11 |  |  |  |  |  |  |  |
| Age | 0 | 0 | 0.01 | 0.01 | 0.01 | 0 | 0 | 0 | 0 | 0 | -0.01 | -0.01 | 0 | 0 | 0.01 | -0.01 | -0.01 | 0 |  |  |  |  |  |  |  |
| **WS2017** |  |  |  |  |  |  |  |  |  |  |  |  |  |  |  |  |  |  |  |  |  |  |  |  |  |
| (Intercept) | 9.95** | 4.95** | 6.46** | 5.75** | 2.97** | 2.53** | 3.38** | 5.59** | 4.67** | 4.17** | 6.74** | 8.27** | 7.79** | 2.54** | 6.51** | 6.49** | 4.75** | 5.13** | 1.37** |  |  |  |  |  |  |
| I(Place/10) | -0.04** | -0.02** | -0.02** | 0 | 0.01* | 0 | 0.02** | 0.01 | 0.02** | -0.01 | 0.03** | 0.08** | 0.03* | -0.01 | 0 | -0.04** | -0.03** | -0.02* | -0.01 |  |  |  |  |  |  |
| Sex: Male | -0.55** | -0.14 | -0.09 | 0.03 | 0.05 | 0.06 | 0.06 | 0.09 | 0.15 | 0.16 | 0.18 | 0.31 | 0.01 | 0.02 | -0.09 | -0.05 | 0.08 | -0.04 | 0.02 |  |  |  |  |  |  |
| Age | 0.02* | 0.01* | 0.01* | 0.01 | 0.01 | 0 | 0.01 | 0 | -0.01 | 0 | -0.02 | -0.01 | -0.01 | 0 | -0.01 | 0 | 0 | 0 | 0 |  |  |  |  |  |  |
| **WS2018** |  |  |  |  |  |  |  |  |  |  |  |  |  |  |  |  |  |  |  |  |  |  |  |  |  |
| (Intercept) | 9.48** | 5.27** | 6.24** | 6.07** | 3.35** | 2.69** | 4.26** | 5.73** | 4.51** | 4.4** | 6.42** | 8.04** | 6.92** | 2.44** | 5.98** | 6.28** | 5.17** | 5.53** | 1.25** |  |  |  |  |  |  |
| I(Place/10) | -0.07** | -0.02* | -0.02** | -0.01 | 0 | -0.01* | 0 | 0 | 0.01** | -0.01* | 0.01 | 0.04** | 0.05** | 0.02 | 0.01 | 0.01 | -0.02** | -0.01 | 0 |  |  |  |  |  |  |
| Sex: Male | -0.27 | -0.19 | -0.08 | 0.13 | 0.14 | 0.08 | 0.14 | 0.04 | 0.13 | 0.08 | 0.12 | 0.15 | 0.03 | 0.09 | -0.17 | 0.03 | -0.17 | -0.14 | 0.01 |  |  |  |  |  |  |
| Age | 0.02* | 0 | 0.01 | 0.01 | 0 | 0 | -0.01 | 0 | -0.01 | -0.01 | -0.01 | -0.01 | 0 | 0.01 | 0 | -0.01 | 0 | 0 | 0 |  |  |  |  |  |  |
| **WS2019** |  |  |  |  |  |  |  |  |  |  |  |  |  |  |  |  |  |  |  |  |  |  |  |  |  |
| (Intercept) | 10.84** | 5.37** | 6.38** | 6.1** | 3.45** | 2.63** | 4.04** | 5.57** | 4.2** | 4.02** | 6.29** | 17.77** | 5.82** | 6.18** | 4.92** | 5.22** | 1.24** |  |  |  |  |  |  |  |  |
| I(Place/10) | -0.05** | -0.02** | -0.02** | -0.01** | 0 | 0 | 0 | 0 | 0.02** | -0.01** | 0.02** | 0.1** | 0.01* | -0.01 | -0.03** | -0.01 | 0 |  |  |  |  |  |  |  |  |
| Sex: Male | -0.41** | -0.24** | -0.14 | -0.05 | 0.11 | -0.02 | -0.04 | -0.03 | 0.11 | 0.08 | 0.21* | 0.43 | -0.13 | 0.1 | -0.12 | 0.13 | 0.05 |  |  |  |  |  |  |  |  |
| Age | 0.01 | 0 | 0.01 | 0 | 0 | 0 | 0 | 0 | 0 | 0 | -0.01* | -0.01 | 0 | 0 | 0.01 | 0 | 0 |  |  |  |  |  |  |  |  |
| **WS2021** |  |  |  |  |  |  |  |  |  |  |  |  |  |  |  |  |  |  |  |  |  |  |  |  |  |
| (Intercept) | 9.85** | 4.77** | 6.15** | 5.78** | 3.01** | 2.45** | 3.95** | 5.67** | 4.53** | 4.34** | 6.8** | 8.46** | 7.51** | 2.77** | 5.74** | 5.87** | 5.31** | 5.7** | 1.36** |  |  |  |  |  |  |
| I(Place/10) | -0.05** | -0.02** | 0 | 0.01 | 0.02* | 0 | 0.02* | 0.02* | 0.03** | 0 | 0.04** | 0.05** | 0.04** | -0.01 | -0.01 | -0.06** | -0.05** | -0.03** | 0 |  |  |  |  |  |  |
| Sex: Male | -0.21 | -0.15 | -0.24 | -0.15 | 0.06 | -0.08 | 0.05 | 0.03 | 0.1 | 0.08 | 0.28 | 0.33 | 0.39* | -0.29 | 0.04 | -0.21 | -0.09 | 0.04 | 0.01 |  |  |  |  |  |  |
| Age | 0 | 0 | 0 | 0.01 | 0.01 | 0.01* | 0 | 0 | 0 | 0 | -0.02* | -0.01 | -0.01 | 0.01 | 0 | 0.01 | -0.01 | -0.01 | 0 |  |  |  |  |  |  |
| **WS2022** |  |  |  |  |  |  |  |  |  |  |  |  |  |  |  |  |  |  |  |  |  |  |  |  |  |
| (Intercept) | 3.71** | 6.45** | 5** | 6.1** | 6.04** | 2.93** | 2.72** | 3.92** | 5.5** | 4.49** | 4.04** | 6.69** | 3.42** | 4.84** | 7.27** | 2.69** | 6.35** | 6.37** | 4.91** | 5.24** | 1.34** |  |  |  |  |
| I(Place/10) | -0.02** | -0.02** | -0.01** | 0 | 0 | 0.01* | 0.01* | 0.01* | 0.01 | 0.02** | -0.01 | 0.03** | 0.03** | 0.01** | 0.03** | 0 | -0.01 | -0.04** | -0.03** | -0.02** | 0 |  |  |  |  |
| Sex: Male | -0.09 | -0.18 | -0.17* | -0.13 | -0.01 | 0.12 | 0.04 | 0.05 | 0.06 | 0.16 | -0.01 | 0.14 | 0.08 | -0.04 | 0 | 0.04 | 0.11 | 0.12 | -0.05 | -0.09 | -0.03 |  |  |  |  |
| Age | 0 | 0 | 0 | 0.01 | 0.01 | 0 | 0 | 0 | 0 | 0 | 0 | -0.01 | -0.01 | 0 | 0 | 0 | -0.01 | 0 | 0 | 0 | 0 |  |  |  |  |

<0.01 = *; <0.001 = **
