# Supplementary material for: Pacing and placing in 161-km ultramarathons: Effects of sex and age
Source: PLoS One. 2025 May 12;20(5):e0322883. doi: 10.1371/journal.pone.0322883 (PMC12068597; doi:10.1371/journal.pone.0322883)
Supplement: S2 Table — (DOCX) [file pone.0322883.s002.docx]

Supplementary Table 2: Linear model results predicting finishing place by sex and age.

| **Race** | **Sex**  Beta (P.value) | **Age** Beta (P.value) |
| --- | --- | --- |
| **HR** |  |  |
| 2012 | -3.18 (0.699) | **1.24 (<0.001)** |
| 2013 | -1.66 (0.851) | **1.39 (<0.001)** |
| 2014 | -17.44 (0.046) | **1.08 (<0.001)** |
| 2015 | -1.56 (0.862) | **1.25 (0.001)** |
| 2016 | -8.32 (0.347) | **1.32 (<0.001)** |
| 2017 | -3.84 (0.649) | **1.70 (<0.001)** |
| 2018 | 0.11 (0.991) | **1.37 (<0.001)** |
| 2021 | 3.17 (0.721) | **2.00 (<0.001)** |
| 2022 | -1.95 (0.787) | **2.10 (<0.001)** |
| **HURT** |  |  |
| 2015 | -2.44 (0.683) | 0.80 (0.002) |
| 2016 | -8.44 (0.118) | 0.59 (0.016) |
| 2017 | -12.04 (0.010) | 0.70 (0.008) |
| 2018 | -14.13 (0.010) | 0.49 (0.070) |
| 2019 | -11.25 (0.057) | 0.79 (0.003) |
| 2020 | **-16.77 (<0.001)** | **0.82 (0.001)** |
| 2022 | -6.43 (0.156) | 0.05 (0.837) |
| **RR** |  |  |
| 2012 | -26.52 (0.012) | **1.54 (<0.001)** |
| 2013 | -27.32 (0.005) | **1.60 (<0.001)** |
| 2014 | **-45.66 (<0.001)** | **2.82 (<0.001)** |
| 2015 | **-43.37 (<0.001)** | **2.44 (<0.001)** |
| 2016 | **-39.08 (<0.001)** | **1.61 (<0.001)** |
| 2017 | **-37.79 (<0.001)** | **1.88 (<0.001)** |
| 2018 | **-37.84 (<0.001)** | 1.07 (0.012) |
| 2019 | **-37.44 (<0.001)** | **2.04 (<0.001)** |
| 2020 | -22.94 (0.028) | 1.60 (0.001) |
| 2021 | **-45.07 (<0.001)** | **2.99 (<0.001)** |
| 2022 | **-30.86 (0.001)** | 1.33 (0.001) |
| **TP** |  |  |
| 2013 | -5.18 (0.560) | 0.62 (0.081) |
| 2014 | -13.11 (0.169) | 0.73 (0.115) |
| 2015 | -18.22 (0.115) | 0.35 (0.444) |
| 2016 | -26.84 (0.036) | 1.49 (0.003) |
| 2017 | **-56.53 (<0.001)** | 1.34 (0.011) |
| 2018 | -23.56 (0.021) | -0.50 (0.278) |
| 2019 | -16.37 (0.139) | 1.85 (0.002) |
| 2020 | -26.98 (0.018) | 1.29 (0.010) |
| 2021 | -16.81 (0.157) | 1.44 (0.009) |
| 2022 | -11.99 (0.227) | 1.44 (0.002) |
| **UTMB** |  |  |
| 2013 | **-144.99 (0.001)** | **14.58 (<0.001)** |
| 2014 | **-143.12 (0.001)** | **11.26 (<0.001)** |
| 2015 | -90.5 (0.029) | **14.63 (<0.001)** |
| 2016 | -104.06 (0.006) | **11.51 (<0.001)** |
| 2017 | -90.52 (0.028) | **16.09 (<0.001)** |
| 2018 | -90.41 (0.025) | **16.44 (<0.001)** |
| 2019 | **-134.86 (<0.001)** | **16.03 (<0.001)** |
| 2021 | -83.85 (0.040) | **19.29 (<0.001)** |
| 2022 | -105.55 (0.015) | **21.01 (<0.001)** |
| **WS** |  |  |
| 2012 | 4.89 (0.707) | **3.89 (<0.001)** |
| 2013 | -6.55 (0.588) | **2.17 (<0.001)** |
| 2014 | -36.88 (0.002) | **3.40 (<0.001)** |
| 2015 | -22.93 (0.025) | **3.74 (<0.001)** |
| 2016 | -16.68 (0.116) | **3.03 (<0.001)** |
| 2017 | -3.01 (0.790) | **2.83 (<0.001)** |
| 2018 | 0.38 (0.972) | **3.16 (<0.001)** |
| 2019 | -29.29 (0.014) | **4.55 (<0.001)** |
| 2021 | -6.59 (0.470) | **3.66 (<0.001)** |
| 2022 | -23.26 (0.031) | **5.18 (<0.001)** |
