# Supplementary material for: Pacing and placing in 161-km ultramarathons: Effects of sex and age
Source: PLoS One. 2025 May 12;20(5):e0322883. doi: 10.1371/journal.pone.0322883 (PMC12068597; doi:10.1371/journal.pone.0322883)
Supplement: S3 Table — (DOCX) [file pone.0322883.s003.docx]

Supplementary Table 3: Generalized Estimating Equation results predicting pace by place.

|  | All segments | | |  | Excluding first segment | | |  | Excluding first and second segment | | |  |
| --- | --- | --- | --- | --- | --- | --- | --- | --- | --- | --- | --- | --- |
|  | Beta | 99.9% CI | P.value |  | Beta | 99.9% CI | P.value |  | Beta | 99.9% CI | P.value | |
| **HURT 2015** |  |  |  |  |  |  |  |  |  |  |  | |
| Place/10 | 0.70 | (0.47, 0.93) | <0.001 |  | 0.79 | (0.52, 1.07) | <0.001 |  | 0.93 | (0.60, 1.25) | <0.001 | |
| Segment | 0.35 | (0.23, 0.48) | <0.001 |  | 0.37 | (0.24, 0.51) | <0.001 |  | 0.39 | (0.23, 0.54) | <0.001 | |
| Sex: Male | -0.22 | (-0.56, 0.12) | 0.035 |  | -0.20 | (-0.58, 0.18) | 0.084 |  | -0.17 | (-0.60, 0.25) | 0.178 | |
| Age | 0.00 | (-0.02, 0.03) | 0.481 |  | 0.00 | (-0.02, 0.03) | 0.679 |  | 0.00 | (-0.03, 0.03) | 0.868 | |
| Place/10 * Segment | 0.01 | (-0.03, 0.04) | 0.383 |  | 0.00 | (-0.04, 0.04) | 0.953 |  | -0.01 | (-0.05, 0.03) | 0.379 | |
| **HURT 2016** |  |  |  |  |  |  |  |  |  |  |  | |
| Place/10 | 0.71 | (0.34, 1.09) | <0.001 |  | 0.87 | (0.42, 1.33) | <0.001 |  | 0.92 | (0.46, 1.38) | <0.001 | |
| Segment | 0.36 | (0.22, 0.49) | <0.001 |  | 0.41 | (0.26, 0.56) | <0.001 |  | 0.38 | (0.23, 0.53) | <0.001 | |
| Sex: Male | -0.23 | (-0.74, 0.28) | 0.132 |  | -0.27 | (-0.81, 0.28) | 0.107 |  | -0.23 | (-0.78, 0.32) | 0.172 | |
| Age | 0.01 | (-0.02, 0.05) | 0.173 |  | 0.01 | (-0.02, 0.05) | 0.252 |  | 0.01 | (-0.03, 0.04) | 0.500 | |
| Place/10 * Segment | 0.01 | (-0.03, 0.05) | 0.271 |  | 0.00 | (-0.05, 0.05) | 0.943 |  | 0.00 | (-0.05, 0.04) | 0.785 | |
| **HURT 2017** |  |  |  |  |  |  |  |  |  |  |  | |
| Place/10 | 0.85 | (0.61, 1.08) | <0.001 |  | 0.89 | (0.61, 1.17) | <0.001 |  | 0.97 | (0.63, 1.32) | <0.001 | |
| Segment | 0.38 | (0.29, 0.47) | <0.001 |  | 0.40 | (0.30, 0.49) | <0.001 |  | 0.39 | (0.29, 0.50) | <0.001 | |
| Sex: Male | 0.01 | (-0.42, 0.45) | 0.933 |  | 0.01 | (-0.45, 0.48) | 0.919 |  | 0.03 | (-0.46, 0.52) | 0.859 | |
| Age | 0.00 | (-0.03, 0.03) | 0.694 |  | 0.00 | (-0.03, 0.03) | 0.732 |  | 0.00 | (-0.04, 0.03) | 0.731 | |
| Place/10 * Segment | 0.00 | (-0.03, 0.02) | 0.655 |  | -0.01 | (-0.04, 0.02) | 0.391 |  | -0.02 | (-0.05, 0.02) | 0.139 | |
| **HURT 2018** |  |  |  |  |  |  |  |  |  |  |  | |
| Place/10 | 0.52 | (0.40, 0.65) | <0.001 |  | 0.61 | (0.46, 0.76) | <0.001 |  | 0.60 | (0.44, 0.76) | <0.001 | |
| Segment | 0.36 | (0.28, 0.44) | <0.001 |  | 0.41 | (0.32, 0.49) | <0.001 |  | 0.36 | (0.27, 0.46) | <0.001 | |
| Sex: Male | 0.04 | (-0.29, 0.37) | 0.701 |  | 0.05 | (-0.30, 0.40) | 0.627 |  | 0.07 | (-0.28, 0.41) | 0.523 | |
| Age | 0.00 | (-0.02, 0.02) | 0.794 |  | 0.00 | (-0.02, 0.02) | 0.943 |  | 0.00 | (-0.02, 0.01) | 0.545 | |
| Place/10 * Segment | 0.00 | (-0.02, 0.02) | 0.833 |  | -0.01 | (-0.03, 0.01) | 0.113 |  | -0.01 | (-0.03, 0.01) | 0.217 | |
| **HURT 2019** |  |  |  |  |  |  |  |  |  |  |  | |
| Place/10 | 0.56 | (0.34, 0.78) | <0.001 |  | 0.60 | (0.36, 0.84) | <0.001 |  | 0.64 | (0.37, 0.91) | <0.001 | |
| Segment | 0.31 | (0.21, 0.42) | <0.001 |  | 0.34 | (0.23, 0.45) | <0.001 |  | 0.34 | (0.22, 0.46) | <0.001 | |
| Sex: Male | -0.08 | (-0.36, 0.19) | 0.326 |  | -0.08 | (-0.38, 0.22) | 0.381 |  | -0.09 | (-0.43, 0.26) | 0.411 | |
| Age | -0.01 | (-0.02, 0.01) | 0.048 |  | -0.01 | (-0.02, 0.01) | 0.057 |  | -0.01 | (-0.02, 0.01) | 0.097 | |
| Place/10 * Segment | 0.01 | (-0.01, 0.04) | 0.064 |  | 0.01 | (-0.02, 0.04) | 0.217 |  | 0.01 | (-0.02, 0.04) | 0.451 | |
| **HURT 2020** |  |  |  |  |  |  |  |  |  |  |  | |
| Place/10 | 0.59 | (0.36, 0.83) | <0.001 |  | 0.67 | (0.41, 0.92) | <0.001 |  | 0.73 | (0.45, 1.02) | <0.001 | |
| Segment | 0.28 | (0.18, 0.39) | <0.001 |  | 0.30 | (0.20, 0.41) | <0.001 |  | 0.30 | (0.19, 0.41) | <0.001 | |
| Sex: Male | -0.04 | (-0.48, 0.41) | 0.780 |  | -0.02 | (-0.49, 0.46) | 0.903 |  | 0.01 | (-0.51, 0.52) | 0.960 | |
| Age | 0.00 | (-0.03, 0.02) | 0.605 |  | 0.00 | (-0.03, 0.02) | 0.571 |  | 0.00 | (-0.03, 0.02) | 0.621 | |
| Place/10 * Segment | 0.01 | (-0.01, 0.04) | 0.098 |  | 0.01 | (-0.02, 0.04) | 0.376 |  | 0.00 | (-0.03, 0.04) | 0.807 | |
| **HURT 2022** |  |  |  |  |  |  |  |  |  |  |  | |
| Place/10 | 0.71 | (0.31, 1.12) | <0.001 |  | 0.69 | (0.24, 1.14) | <0.001 |  | 0.73 | (0.22, 1.25) | <0.001 | |
| Segment | 0.29 | (0.15, 0.44) | <0.001 |  | 0.29 | (0.13, 0.45) | <0.001 |  | 0.27 | (0.09, 0.46) | <0.001 | |
| Sex: Male | 0.06 | (-0.35, 0.47) | 0.635 |  | 0.06 | (-0.36, 0.47) | 0.653 |  | 0.07 | (-0.35, 0.50) | 0.567 | |
| Age | 0.01 | (-0.01, 0.03) | 0.258 |  | 0.01 | (-0.01, 0.03) | 0.316 |  | 0.01 | (-0.01, 0.03) | 0.381 | |
| Place/10 * Segment | 0.01 | (-0.04, 0.06) | 0.520 |  | 0.01 | (-0.04, 0.07) | 0.467 |  | 0.01 | (-0.05, 0.07) | 0.656 | |
| **RR 2012** |  |  |  |  |  |  |  |  |  |  |  | |
| Place/10 | 0.09 | (0.04, 0.14) | <0.001 |  | 0.11 | (0.03, 0.18) | <0.001 |  | 0.16 | (0.03, 0.30) | <0.001 | |
| Segment | 0.71 | (0.53, 0.89) | <0.001 |  | 0.85 | (0.61, 1.08) | <0.001 |  | 0.99 | (0.65, 1.33) | <0.001 | |
| Sex: Male | -0.06 | (-0.21, 0.08) | 0.151 |  | 0.03 | (-0.15, 0.21) | 0.57 |  | 0.11 | (-0.15, 0.37) | 0.156 | |
| Age | 0.00 | (0, 0.01) | 0.033 |  | 0.00 | (-0.01, 0.01) | 0.311 |  | 0.00 | (-0.01, 0.01) | 0.749 | |
| Place/10 * Segment | 0.04 | (0.02, 0.05) | <0.001 |  | 0.03 | (0.01, 0.06) | <0.001 |  | 0.02 | (-0.01, 0.06) | 0.042 | |
| **RR 2013** |  |  |  |  |  |  |  |  |  |  |  | |
| Place/10 | 0.09 | (0.06, 0.13) | <0.001 |  | 0.12 | (0.05, 0.18) | <0.001 |  | 0.16 | (0.05, 0.26) | <0.001 | |
| Segment | 0.73 | (0.57, 0.89) | <0.001 |  | 0.83 | (0.61, 1.06) | <0.001 |  | 0.89 | (0.56, 1.23) | <0.001 | |
| Sex: Male | -0.02 | (-0.13, 0.09) | 0.539 |  | 0.01 | (-0.13, 0.15) | 0.817 |  | 0.05 | (-0.16, 0.26) | 0.420 | |
| Age | 0.00 | (-0.01, 0.01) | 0.832 |  | 0.00 | (-0.01, 0) | 0.236 |  | -0.01 | (-0.02, 0.00) | 0.039 | |
| Place/10 * Segment | 0.03 | (0.02, 0.05) | <0.001 |  | 0.03 | (0.01, 0.05) | <0.001 |  | 0.02 | (-0.01, 0.05) | 0.016 | |
| **RR 2014** |  |  |  |  |  |  |  |  |  |  |  | |
| Place/10 | 0.09 | (0.07, 0.12) | <0.001 |  | 0.13 | (0.09, 0.16) | <0.001 |  | 0.21 | (0.14, 0.28) | <0.001 | |
| Segment | 0.71 | (0.58, 0.85) | <0.001 |  | 0.85 | (0.67, 1.04) | <0.001 |  | 1.07 | (0.79, 1.34) | <0.001 | |
| Sex: Male | -0.01 | (-0.2, 0.17) | 0.799 |  | 0.04 | (-0.19, 0.26) | 0.606 |  | 0.03 | (-0.24, 0.31) | 0.683 | |
| Age | 0.01 | (0, 0.02) | 0.029 |  | 0.01 | (-0.01, 0.02) | 0.149 |  | 0.01 | (-0.01, 0.02) | 0.219 | |
| Place/10 * Segment | 0.03 | (0.02, 0.03) | <0.001 |  | 0.02 | (0, 0.03) | <0.001 |  | 0.00 | (-0.02, 0.02) | 0.772 | |
| **RR 2015** |  |  |  |  |  |  |  |  |  |  |  | |
| Place/10 | 0.09 | (0.06, 0.12) | <0.001 |  | 0.11 | (0.06, 0.15) | <0.001 |  | 0.18 | (0.10, 0.26) | <0.001 | |
| Segment | 0.64 | (0.49, 0.78) | <0.001 |  | 0.81 | (0.62, 1.01) | <0.001 |  | 1.05 | (0.77, 1.33) | <0.001 | |
| Sex: Male | 0.08 | (-0.12, 0.27) | 0.186 |  | 0.11 | (-0.12, 0.34) | 0.106 |  | 0.15 | (-0.16, 0.46) | 0.106 | |
| Age | 0.00 | (0, 0.01) | 0.041 |  | 0.00 | (-0.01, 0.01) | 0.184 |  | 0.00 | (-0.01, 0.01) | 0.785 | |
| Place/10 * Segment | 0.04 | (0.03, 0.05) | <0.001 |  | 0.04 | (0.02, 0.05) | <0.001 |  | 0.02 | (0.00, 0.04) | 0.006 | |
| **RR 2016** |  |  |  |  |  |  |  |  |  |  |  | |
| Place/10 | 0.09 | (0.05, 0.12) | <0.001 |  | 0.12 | (0.06, 0.18) | <0.001 |  | 0.21 | (0.10, 0.32) | <0.001 | |
| Segment | 0.74 | (0.58, 0.9) | <0.001 |  | 0.92 | (0.7, 1.14) | <0.001 |  | 1.17 | (0.84, 1.50) | <0.001 | |
| Sex: Male | 0.05 | (-0.11, 0.22) | 0.284 |  | 0.13 | (-0.08, 0.33) | 0.040 |  | 0.19 | (-0.09, 0.46) | 0.025 | |
| Age | 0.00 | (0, 0.01) | 0.191 |  | 0.00 | (-0.01, 0.01) | 0.788 |  | 0.00 | (-0.01, 0.01) | 0.828 | |
| Place/10 * Segment | 0.03 | (0.02, 0.05) | <0.001 |  | 0.03 | (0.01, 0.05) | <0.001 |  | 0.00 | (-0.03, 0.03) | 0.654 | |
| **RR 2017** |  |  |  |  |  |  |  |  |  |  |  | |
| Place/10 | 0.10 | (0.06, 0.14) | <0.001 |  | 0.12 | (0.05, 0.19) | <0.001 |  | 0.20 | (0.06, 0.34) | <0.001 | |
| Segment | 0.73 | (0.57, 0.89) | <0.001 |  | 0.91 | (0.69, 1.13) | <0.001 |  | 1.17 | (0.82, 1.52) | <0.001 | |
| Sex: Male | -0.07 | (-0.24, 0.11) | 0.195 |  | -0.01 | (-0.21, 0.19) | 0.849 |  | 0.03 | (-0.26, 0.33) | 0.697 | |
| Age | 0.00 | (0, 0.01) | 0.133 |  | 0.00 | (-0.01, 0.01) | 0.238 |  | 0.00 | (-0.01, 0.02) | 0.740 | |
| Place/10 * Segment | 0.04 | (0.03, 0.06) | <0.001 |  | 0.04 | (0.01, 0.06) | <0.001 |  | 0.02 | (-0.02, 0.06) | 0.140 | |
| **RR 2018** |  |  |  |  |  |  |  |  |  |  |  | |
| Place/10 | 0.12 | (0.06, 0.17) | <0.001 |  | 0.19 | (0.09, 0.29) | <0.001 |  | 0.47 | (0.27, 0.67) | <0.001 | |
| Segment | 1.16 | (0.91, 1.4) | <0.001 |  | 1.51 | (1.12, 1.9) | <0.001 |  | 2.07 | (1.38, 2.76) | <0.001 | |
| Sex: Male | 0.03 | (-0.17, 0.23) | 0.646 |  | 0.08 | (-0.18, 0.34) | 0.318 |  | 0.03 | (-0.41, 0.47) | 0.816 | |
| Age | 0.00 | (0, 0.01) | 0.130 |  | 0.00 | (-0.01, 0.01) | 0.446 |  | 0.00 | (-0.02, 0.02) | 0.601 | |
| Place/10 * Segment | 0.04 | (0.01, 0.06) | <0.001 |  | 0.02 | (-0.02, 0.05) | 0.191 |  | -0.06 | (-0.12, 0.00) | 0.001 | |
| **RR 2019** |  |  |  |  |  |  |  |  |  |  |  | |
| Place/10 | 0.12 | (0.07, 0.17) | <0.001 |  | 0.19 | (0.10, 0.28) | <0.001 |  | 0.51 | (0.32, 0.71) | <0.001 | |
| Segment | 1.24 | (1.00, 1.48) | <0.001 |  | 1.58 | (1.23, 1.94) | <0.001 |  | 2.22 | (1.58, 2.86) | <0.001 | |
| Sex: Male | 0.00 | (-0.15, 0.16) | 0.970 |  | 0.09 | (-0.15, 0.34) | 0.200 |  | 0.19 | (-0.25, 0.64) | 0.156 | |
| Age | 0.00 | (-0.01, 0.01) | 0.548 |  | 0.00 | (-0.01, 0.01) | 0.96 |  | -0.01 | (-0.03, 0.02) | 0.368 | |
| Place/10 * Segment | 0.03 | (0.01, 0.05) | 0.00 |  | 0.01 | (-0.02, 0.05) | 0.22 |  | -0.07 | (-0.14, -0.01) | <0.001 | |
| **RR 2020** |  |  |  |  |  |  |  |  |  |  |  | |
| Place/10 | 0.08 | (0.05, 0.11) | <0.001 |  | 0.11 | (0.05, 0.17) | <0.001 |  | 0.26 | (0.13, 0.39) | <0.001 | |
| Segment | 1.07 | (0.89, 1.24) | <0.001 |  | 1.30 | (1.04, 1.57) | <0.001 |  | 1.58 | (1.12, 2.04) | <0.001 | |
| Sex: Male | -0.08 | (-0.21, 0.05) | 0.042 |  | -0.02 | (-0.2, 0.16) | 0.743 |  | 0.03 | (-0.28, 0.35) | 0.722 | |
| Age | 0.01 | (0.00, 0.01) | 0.003 |  | 0.01 | (0.00, 0.02) | 0.009 |  | 0.01 | (-0.01, 0.02) | 0.058 | |
| Place/10 * Segment | 0.03 | (0.02, 0.05) | <0.001 |  | 0.02 | (0.00, 0.05) | <0.001 |  | -0.02 | (-0.06, 0.02) | 0.172 | |
| **RR 2021** |  |  |  |  |  |  |  |  |  |  |  | |
| Place/10 | 0.07 | (0.03, 0.10) | <0.001 |  | 0.08 | (0.02, 0.14) | <0.001 |  | 0.14 | (0.03, 0.24) | <0.001 | |
| Segment | 0.58 | (0.44, 0.71) | <0.001 |  | 0.66 | (0.47, 0.85) | <0.001 |  | 0.79 | (0.51, 1.07) | <0.001 | |
| Sex: Male | -0.01 | (-0.10, 0.08) | 0.701 |  | -0.01 | (-0.14, 0.11) | 0.733 |  | -0.03 | (-0.25, 0.19) | 0.634 | |
| Age | 0.00 | (-0.01, 0.01) | 0.971 |  | 0.00 | (-0.01, 0.00) | 0.283 |  | 0.00 | (-0.02, 0.01) | 0.179 | |
| Place/10 * Segment | 0.04 | (0.03, 0.05) | <0.001 |  | 0.04 | (0.02, 0.06) | <0.001 |  | 0.02 | (0.00, 0.05) | 0.005 | |
| **RR 2022** |  |  |  |  |  |  |  |  |  |  |  | |
| Place/10 | 0.09 | (0.04, 0.15) | <0.001 |  | 0.10 | (0.02, 0.19) | <0.001 |  | 0.21 | (0.05, 0.37) | <0.001 | |
| Segment | 0.68 | (0.51, 0.86) | <0.001 |  | 0.79 | (0.55, 1.03) | <0.001 |  | 1.03 | (0.66, 1.41) | <0.001 | |
| Sex: Male | -0.02 | (-0.14, 0.11) | 0.652 |  | 0.05 | (-0.11, 0.21) | 0.313 |  | 0.11 | (-0.13, 0.36) | 0.115 | |
| Age | 0.00 | (0.00, 0.01) | 0.011 |  | 0.00 | (-0.01, 0.01) | 0.425 |  | 0.00 | (-0.01, 0.01) | 0.632 | |
| Place/10 * Segment | 0.05 | (0.03, 0.07) | <0.001 |  | 0.05 | (0.02, 0.08) | <0.001 |  | 0.03 | (-0.02, 0.07) | 0.049 | |
| **TP 2013** |  |  |  |  |  |  |  |  |  |  |  | |
| Place/10 | 0.25 | (0.12, 0.39) | <0.001 |  | 0.33 | (0.14, 0.51) | <0.001 |  | 0.43 | (0.19, 0.68) | <0.001 | |
| Segment | 0.42 | (0.33, 0.52) | <0.001 |  | 0.46 | (0.34, 0.57) | <0.001 |  | 0.46 | (0.32, 0.60) | <0.001 | |
| Sex: Male | -0.03 | (-0.25, 0.19) | 0.676 |  | -0.01 | (-0.26, 0.23) | 0.874 |  | 0.01 | (-0.28, 0.30) | 0.912 | |
| Age | 0.00 | (0.00, 0.01) | 0.130 |  | 0.00 | (0.00, 0.01) | 0.133 |  | 0.00 | (-0.01, 0.01) | 0.180 | |
| Place/10 * Segment | 0.03 | (0.01, 0.06) | <0.001 |  | 0.02 | (-0.01, 0.05) | 0.008 |  | 0.01 | (-0.02, 0.05) | 0.310 | |
| **TP 2014** |  |  |  |  |  |  |  |  |  |  |  | |
| Place/10 | 0.12 | (0.06, 0.17) | <0.001 |  | 0.15 | (0.07, 0.24) | <0.001 |  | 0.21 | (0.08, 0.34) | <0.001 | |
| Segment | 0.66 | (0.53, 0.78) | <0.001 |  | 0.61 | (0.44, 0.77) | <0.001 |  | 0.60 | (0.37, 0.83) | <0.001 | |
| Sex: Male | -0.02 | (-0.09, 0.06) | 0.494 |  | 0.04 | (-0.09, 0.17) | 0.333 |  | 0.02 | (-0.15, 0.19) | 0.689 | |
| Age | 0.00 | (0.00, 0.01) | 0.343 |  | 0.00 | (-0.01, 0.01) | 0.333 |  | 0.00 | (-0.01, 0.01) | 0.243 | |
| Place/10 * Segment | 0.04 | (0.02, 0.05) | <0.001 |  | 0.03 | (0.01, 0.05) | <0.001 |  | 0.02 | (-0.01, 0.05) | 0.065 | |
| **TP 2015** |  |  |  |  |  |  |  |  |  |  |  | |
| Place/10 | 0.12 | (0.08, 0.17) | <0.001 |  | 0.16 | (0.09, 0.22) | <0.001 |  | 0.19 | (0.09, 0.29) | <0.001 | |
| Segment | 0.41 | (0.31, 0.51) | <0.001 |  | 0.34 | (0.21, 0.46) | <0.001 |  | 0.37 | (0.20, 0.54) | <0.001 | |
| Sex: Male | 0.13 | (-0.04, 0.3) | 0.012 |  | 0.18 | (-0.02, 0.39) | 0.003 |  | 0.14 | (-0.1, 0.39) | 0.057 | |
| Age | 0.00 | (-0.01, 0.01) | 0.275 |  | 0.00 | (-0.01, 0.01) | 0.421 |  | 0.00 | (-0.01, 0.01) | 0.613 | |
| Place/10 * Segment | 0.02 | (0.01, 0.03) | <0.001 |  | 0.02 | (0.00, 0.03) | <0.001 |  | 0.01 | (-0.01, 0.03) | 0.082 | |
| **TP 2016** |  |  |  |  |  |  |  |  |  |  |  | |
| Place/10 | 0.14 | (0.10, 0.18) | <0.001 |  | 0.21 | (0.14, 0.28) | <0.001 |  | 0.36 | (0.25, 0.48) | <0.001 | |
| Segment | 0.48 | (0.38, 0.59) | <0.001 |  | 0.35 | (0.20, 0.50) | <0.001 |  | 0.22 | (-0.01, 0.46) | <0.001 | |
| Sex: Male | 0.03 | (-0.17, 0.22) | 0.660 |  | 0.03 | (-0.22, 0.28) | 0.653 |  | 0.01 | (-0.30, 0.33) | 0.878 | |
| Age | 0.00 | (-0.01, 0.00) | 0.237 |  | 0.00 | (-0.01, 0.00) | 0.084 |  | -0.01 | (-0.02, 0.00) | 0.009 | |
| Place/10 * Segment | 0.02 | (0.0, 0.03) | <0.001 |  | 0.00 | (-0.02, 0.02) | 0.696 |  | -0.03 | (-0.05, 0.00) | <0.001 | |
| **TP 2017** |  |  |  |  |  |  |  |  |  |  |  | |
| Place/10 | 0.17 | (0.14, 0.2) | <0.001 |  | 0.24 | (0.19, 0.30) | <0.001 |  | 0.35 | (0.27, 0.44) | <0.001 | |
| Segment | 0.55 | (0.43, 0.68) | <0.001 |  | 0.42 | (0.25, 0.58) | <0.001 |  | 0.20 | (-0.03, 0.44) | 0.005 | |
| Sex: Male | 0.16 | (-0.06, 0.39) | 0.018 |  | 0.23 | (-0.06, 0.52) | 0.008 |  | 0.26 | (-0.13, 0.65) | 0.026 | |
| Age | 0.00 | (-0.01, 0.01) | 0.867 |  | 0.00 | (-0.02, 0.01) | 0.308 |  | -0.01 | (-0.02, 0.01) | 0.083 | |
| Place/10 * Segment | 0.01 | (0.00, 0.02) | 0.003 |  | -0.01 | (-0.02, 0.01) | 0.115 |  | -0.03 | (-0.05, -0.01) | <0.001 | |
| **TP 2018** |  |  |  |  |  |  |  |  |  |  |  | |
| Place/10 | 0.07 | (0.03, 0.11) | <0.001 |  | 0.08 | (0.02, 0.15) | <0.001 |  | 0.06 | (-0.03, 0.16) | 0.035 | |
| Segment | 0.55 | (0.43, 0.66) | <0.001 |  | 0.41 | (0.27, 0.56) | <0.001 |  | 0.12 | (-0.07, 0.31) | 0.038 | |
| Sex: Male | 0.03 | (-0.09, 0.15) | 0.383 |  | 0.08 | (-0.08, 0.23) | 0.093 |  | 0.11 | (-0.13, 0.34) | 0.140 | |
| Age | 0.00 | (-0.01, 0.01) | 0.906 |  | 0.00 | (-0.01, 0.01) | 0.427 |  | -0.01 | (-0.02, 0.01) | 0.109 | |
| Place/10 * Segment | 0.03 | (0.02, 0.04) | <0.001 |  | 0.03 | (0.01, 0.05) | <0.001 |  | 0.03 | (0.01, 0.06) | <0.001 | |
| **TP 2019** |  |  |  |  |  |  |  |  |  |  |  | |
| Place/10 | 0.08 | (0.05, 0.11) | <0.001 |  | 0.11 | (0.08, 0.15) | <0.001 |  | 0.14 | (0.09, 0.19) | <0.001 | |
| Segment | 0.33 | (0.26, 0.41) | <0.001 |  | 0.31 | (0.23, 0.40) | <0.001 |  | 0.22 | (0.12, 0.33) | <0.001 | |
| Sex: Male | 0.01 | (-0.13, 0.15) | 0.825 |  | 0.03 | (-0.14, 0.19) | 0.555 |  | 0.06 | (-0.15, 0.26) | 0.350 | |
| Age | 0.00 | (-0.01, 0.01) | 0.731 |  | 0.00 | (-0.01, 0.01) | 0.559 |  | 0.00 | (-0.01, 0.01) | 0.288 | |
| Place/10 * Segment | 0.02 | (0.01, 0.02) | <0.001 |  | 0.01 | (0.00, 0.02) | <0.001 |  | 0.01 | (0.00, 0.02) | 0.006 | |
| **TP 2020** |  |  |  |  |  |  |  |  |  |  |  | |
| Place/10 | 0.13 | (0.09, 0.17) | <0.001 |  | 0.18 | (0.13, 0.24) | <0.001 |  | 0.25 | (0.18, 0.33) | <0.001 | |
| Segment | 0.21 | (0.11, 0.31) | <0.001 |  | 0.14 | (0.02, 0.27) | <0.001 |  | 0.01 | (-0.14, 0.15) | 0.891 | |
| Sex: Male | 0.11 | (-0.08, 0.29) | 0.059 |  | 0.16 | (-0.05, 0.37) | 0.013 |  | 0.20 | (-0.06, 0.47) | 0.012 | |
| Age | 0.00 | (-0.01, 0.02) | 0.266 |  | 0.00 | (-0.01, 0.02) | 0.360 |  | 0.00 | (-0.01, 0.02) | 0.501 | |
| Place/10 * Segment | 0.02 | (0.01, 0.03) | <0.001 |  | 0.01 | (0.00, 0.02) | 0.013 |  | 0.00 | (-0.02, 0.01) | 0.621 | |
| **TP 2021** |  |  |  |  |  |  |  |  |  |  |  | |
| Place/10 | 0.17 | (0.13, 0.2) | <0.001 |  | 0.20 | (0.16, 0.24) | <0.001 |  | 0.23 | (0.17, 0.28) | <0.001 | |
| Segment | 0.29 | (0.22, 0.36) | <0.001 |  | 0.27 | (0.19, 0.35) | <0.001 |  | 0.27 | (0.18, 0.36) | <0.001 | |
| Sex: Male | -0.05 | (-0.26, 0.16) | 0.414 |  | -0.04 | (-0.28, 0.2) | 0.572 |  | -0.02 | (-0.29, 0.25) | 0.807 | |
| Age | 0.00 | (-0.01, 0.02) | 0.522 |  | 0.00 | (-0.01, 0.02) | 0.522 |  | 0.00 | (-0.01, 0.02) | 0.715 | |
| Place/10 * Segment | 0.01 | (0, 0.02) | <0.001 |  | 0.01 | (0, 0.01) | 0.051 |  | 0.00 | (-0.01, 0.01) | 0.491 | |
| **TP 2022** |  |  |  |  |  |  |  |  |  |  |  | |
| Place/10 | 0.10 | (0.08, 0.13) | <0.001 |  | 0.12 | (0.09, 0.15) | <0.001 |  | 0.14 | (0.10, 0.18) | <0.001 | |
| Segment | 0.24 | (0.19, 0.29) | <0.001 |  | 0.21 | (0.16, 0.27) | <0.001 |  | 0.17 | (0.11, 0.22) | <0.001 | |
| Sex: Male | 0.08 | (-0.08, 0.24) | 0.087 |  | 0.11 | (-0.07, 0.28) | 0.042 |  | 0.14 | (-0.06, 0.33) | 0.019 | |
| Age | 0.00 | (-0.01, 0.01) | 0.765 |  | 0.00 | (-0.01, 0.01) | 0.867 |  | 0.00 | (-0.01, 0.01) | 0.965 | |
| Place/10 * Segment | 0.01 | (0.01, 0.02) | <0.001 |  | 0.01 | (0.01, 0.02) | <0.001 |  | 0.01 | (0.00, 0.02) | <0.001 | |
